# Linking oral microbiota to periodontitis and hypertension unveils that *Filifactor alocis* aggravates hypertension via infiltration of interferon-γ^+^ T cells

**DOI:** 10.1128/msystems.00084-25

**Published:** 2025-05-21

**Authors:** Jun Zhang, Bo-Yan Chen, Meng-Fan Zhi, Wen-Zhen Lin, Yu-Lin Li, Hui-Lin Ye, Shuo Xu, Hong Zhu, Lu-Jun Zhou, Lin-Juan Du, Xiao-Qian Meng, Yan Liu, Qiang Feng, Sheng-Zhong Duan

**Affiliations:** 1 Department of Endodontics, Shanghai Ninth People’s Hospital, College of Stomatology, Shanghai Jiao Tong University School of Medicine 12474, Shanghai, Shanghai, China; 2 Laboratory of Oral Microbiota and Systemic Diseases, Shanghai Ninth People’s Hospital, College of Stomatology, Shanghai Jiao Tong University School of Medicine; National Center for Stomatology; National Clinical Research Center for Oral Diseases; Shanghai Key Laboratory of Stomatology 56694, Shanghai, Shanghai, China; 3 Stomatology Hospital, School of Stomatology, and Institute of Translational Medicine, Zhejiang University School of Medicine, Zhejiang Provincial Clinical Research Center for Oral Diseases, Key Laboratory of Oral Biomedical Research of Zhejiang Province, Cancer Center of Zhejiang University, Engineering Research Center of Oral Biomaterials and Devices of Zhejiang Province 26441 , Hangzhou, Zhejiang, China; 4 Department of Human Microbiome, School and Hospital of Stomatology, Cheeloo College of Medicine, Shandong University & Shandong Key Laboratory of Oral Tissue Regeneration & Shandong Engineering Laboratory for Dental Materials and Oral Tissue Regeneration & Shandong Provincial Clinical Research Center for Oral Diseases 12589, Jinan, Shandong, China; 5 Shandong University-BOP Joint Oral Microbiome Laboratory, Jinan, Shandong, China; Southern Medical University, Guangzhou, Guandong, China

**Keywords:** periodontitis, hypertension, oral microbiota, *Filifactor alocis*, IFNγ^+ ^T cell

## Abstract

Periodontal disease (PD), an inflammatory disease initiated by oral microbiota, may aggravate hypertension (HTN). Few studies were employed to characterize the oral microbiota in hypertensive patients with periodontitis. To investigate the interplay between oral microbiota and hypertension in individuals with periodontitis, we initiated a metagenomic sequencing study on subgingival plaque and saliva samples sourced from HTN patients and those with hypertension and periodontitis (PDHTN). Our primary objective was to characterize species serving as pivotal links (bridge species) in exacerbating hypertension induced by periodontal disease. Within subgingival plaque and saliva specimens, we pinpointed 31 and 28 bridge species, respectively. Furthermore, we noted a decrease in the abundance of nitrate-reducing bacteria, such as *Actinomyces* spp., *Rothia* spp., and *Veillonella* spp., in PDHTN samples. Employing network analysis, we distinguished distinct polymicrobial clusters within the two patient groups. These bridge species coalesced into polymicrobial clusters, revealing intricate symbiotic and competitive relationships. To substantiate our findings, we leveraged an angiotensin II-infused animal model of ligature-induced periodontitis (LIP), confirming the contributory role of *Filifactor alocis*—a selectively analyzed subgingival bridge species—in exacerbating hypertension and upregulating the frequency of renal CD4^+^IFNγ^+^ and CD8^+^IFNγ^+^ T cells. Our study screened a list of species linking PD and HTN. PD may aggravate HTN by decreasing the abundance of nitrate-reducing bacteria and increasing the abundance of pathogens. Using an animal model, we demonstrated that *F. alocis* aggravates HTN via the accumulation of IFNγ^+^ T cells in the kidneys.

**IMPORTANCE:**

Both periodontal disease and hypertension are widely prevalent all over the world. PD may aggravate the development of HTN via oral microbiota. However, few studies were employed to characterize the oral microbiota in hypertensive patients with periodontitis. Here, the present study profiled the oral microbiota in hypertensive participants with periodontitis. We found that the depleted abundance of nitrate-reducing bacteria and the enriched abundance of pathogens. Finally, we validated the role of *Filifactor alocis* in exacerbating HTN via infiltration of IFNγ^+^ T cells in mice kidneys. Our study improved the understanding of oral microbiota linking PD and HTN.

## INTRODUCTION

Hypertension (HTN) constitutes a pivotal factor driving the global cardiovascular disease burden and overarching mortality rates. Among individuals with systolic blood pressure (SBP) exceeding 140 mmHg, a shocking annual mortality estimate of 106.3 per 100,000 individuals was projected in 2015, as documented (1). Despite the availability of various treatments, essential hypertension continues to be poorly managed (2). This suggests that the fundamental mechanisms of HTN still require further exploration, albeit extensive research has been conducted for more than a century (3).

Periodontitis (PD), an oral inflammatory disorder triggered by bacterial activity, not only erodes tooth-supporting structures but also exerts profound impacts on systemic health (4). Accumulating evidence has illuminated the intricate nexus between PD and HTN, with a retrospective study (5) revealing a mean SBP increase of approximately 2.3–3 mmHg among hypertensive patients concurrently suffering from PD. Consistent with our prior endeavors and numerous other studies, it has been established that hypertensive individuals with PD exhibit elevated blood pressure levels, as compared to those without PD (6, 7). Moreover, a randomized controlled trial (8) underscored a more pronounced decrease in BP among PD patients receiving intensive periodontal therapy, as opposed to those on standard periodontal care. Notably, Tonetti et al. (9) documented improved oral health and endothelial function—a vital regulator of BP—following 6 months of intensive periodontal treatment in PD patients. An animal model study conducted by Xu et al. (10) definitively showed that ligature-induced periodontitis (LIP) intensifies angiotensin II-mediated hypertension in mice. Collectively, these studies underscore the aggravating influence of PD on HTN.

PD arises from the intricate interplay of polymicrobial synergy and dysbiosis (11), underscoring the critical role of microbiota within periodontal tissue. Multiple studies (12, 13) have suggested an association between oral microbiota and HTN. A study (14) shows that increased subgingival bacteria, such as *Aggregatibacter actinomycetemcomitans, Porphyromonas gingivalis, Tannerella forsythia,* and *Treponema denticola* (etiologic bacterial burden from periodontal infection), have a strong positive association with the morbidity of hypertension. Accordingly, the highest level of etiologic bacterial burden has an increased SBP and DBP when compared to the lowest (14). Another study demonstrates that the antigen from *P. gingivalis* can aggravate HTN (15). It is plausible to hypothesize that periodontal disease may aggravate hypertension via altered oral microbiota. Thus, it is essential to decipher the alteration of oral microbiota in hypertensive patients with periodontal disease.

In this context, we undertake an exhaustive species-level analysis of the oral microbiota. Our investigation aims to unravel microbial mediators potentially linking PD and HTN—bridge species capable of amplifying the detrimental impact of PD on BP. Subsequently, an animal study was conducted, reinforcing the finding that *Filifactor alocis*, a subgingival bridge species, can elevate BP amidst periodontal disease.

## RESULTS

### Alterations in the oral microbiota diversity in hypertensive participants with PD

In total, 57 patients (Table S1), 14 healthy participants (healthy control, HC), 8 participants with periodontal disease (PD), 16 hypertensive participants (HTN), and 19 patients with hypertension and periodontitis (PDHTN), were included in this study. Among the individuals in the cohort, 23 participants were men and 34 were women. 86% of healthy participants, 88% of PD patients, 94% of HTN patients, and 90% of PDHTN were more than 60 years old. Systolic blood pressure (SBP; 120 ± 13.5 vs 125 ± 9.75 vs 125 ± 16.8 vs 134 ± 11, *P* = 0.0414) and diastolic blood pressure (DBP; 74.5 ± 6.28 vs 77.9 ± 8.32 vs 75.1 ± 7.58 vs 79.9 ± 8.02, *P* = 0.147) were higher in PDHTN when compared with the other three groups (Table S1).

**Fig 1:**
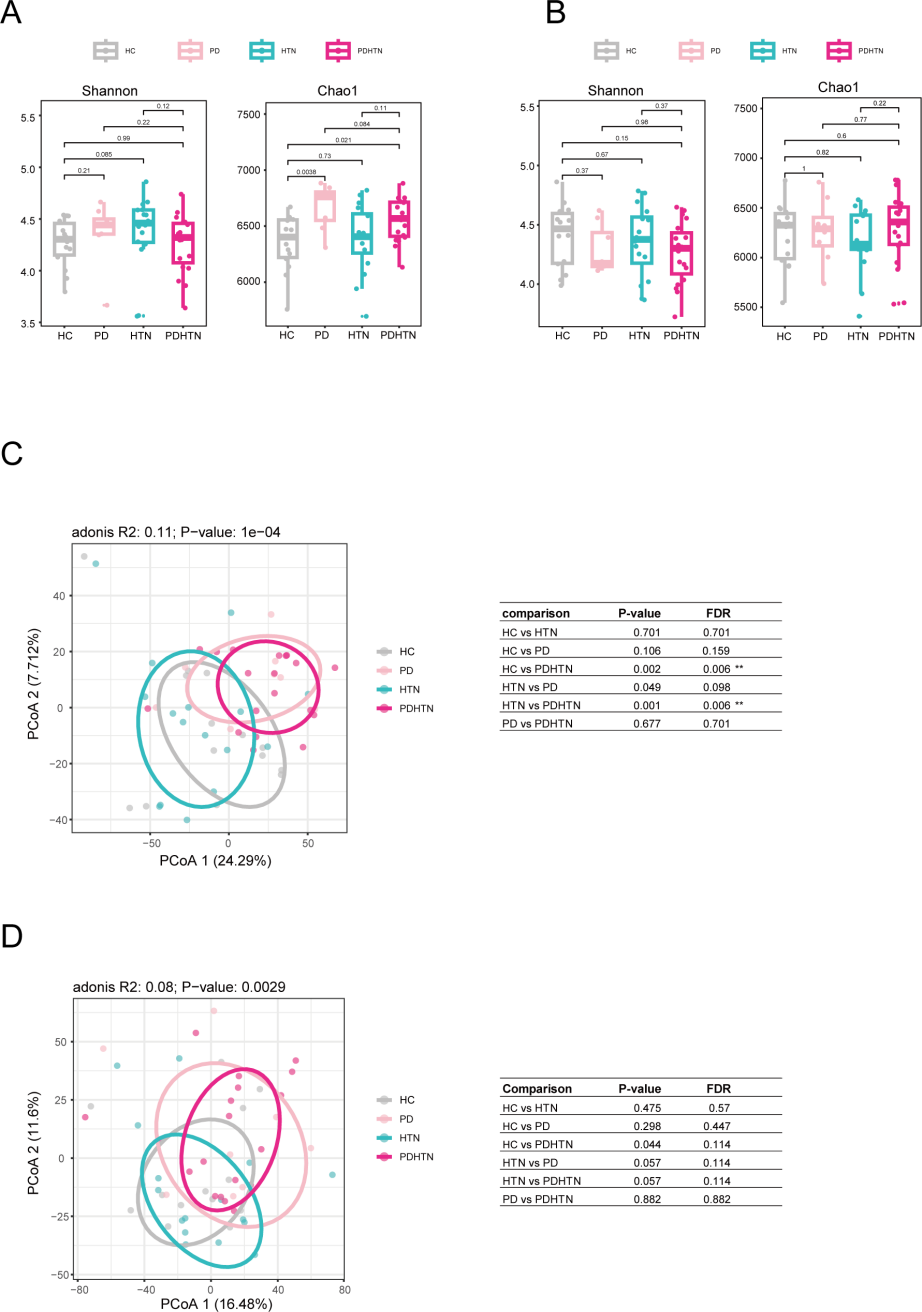
Alterations of the diversity in oral microbiota of participants with PD and HTN. (A, B) Chao1 and Shannon index of microbiota in subgingival plaques (**A**) and saliva (**B**) of healthy participants, participants with PD, participants with HTN, and participants with PDHTN. (C, D) Principal component analysis (PCoA) and the paired *P* value (right table) based on Aitchison distances of microbiota in subgingival plaques (**C**) and saliva (**D**) of four groups. *n* = 14:8:16:19 for healthy, PD, HTN, and PDHTN in subgingival plaques and saliva. Wilcoxon rank-sum test was used for statistical analysis in A and B, and permutational multivariate analysis of variance in panels C and D.

The Chao1 richness and Shannon indices analysis simultaneously showed that the microbial diversity of subgingival plaques and saliva did not show a significant difference among the four groups, except the Chao1 index between HC and PDHTN (Fig. 1A and B). Principal component analysis (PCoA) based on Aitchison distances was performed to assess beta diversity in subgingival plaques and saliva samples. The statistical results showed that the interindividual differences were significantly different among the four groups in both subgingival plaques (Fig. 1C; *P* < 1e−4) and saliva (Fig. 1D; *P* < 0.0029). When compared to HC or HTN groups, PDHTN showed statistically significant differences in subgingival plaques.

### Identification and characterization of bridge species

**Fig 2:**
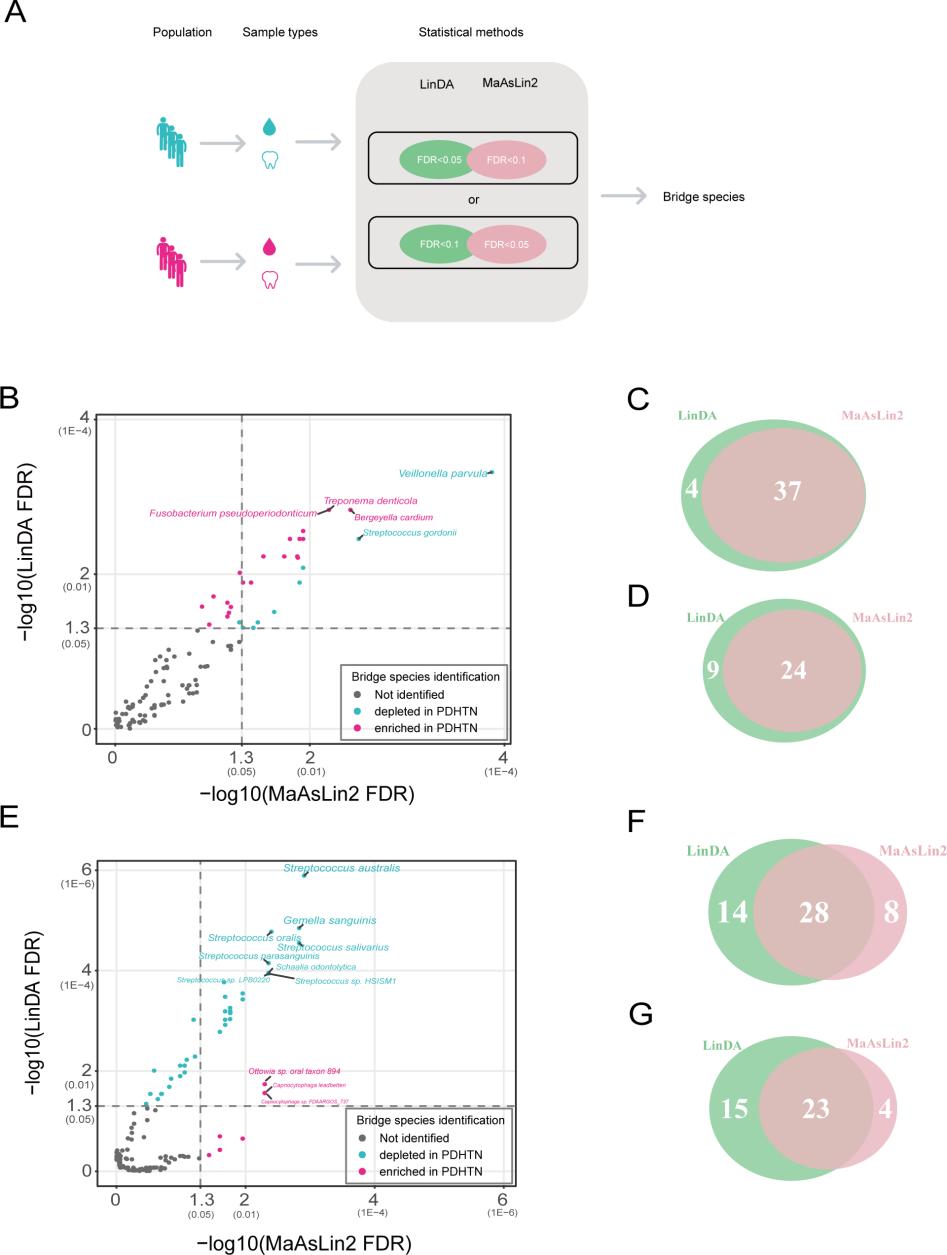
Identification of bridge species. (A) Visual summary of the two statistical methods to identify bridge species. (B) Identification of subgingival bridge species (bridge species, namely species associated with the HTN-aggravating effect of PD). A total of 113 species (selected by mean relative abundance > 0.001) in subgingival plaques were calculated via two methods (LinDA and MaAsLin2) to define the bridge species with FDR (false discovery rate) of <0.05 by one method and FDR < 0.1 by the other. 31 subgingival bridge species were identified, including 9 depleted and 22 enriched in PDHTN. The *X*-axis shows the −log10 of FDR obtained by MaAsLin2, and the *Y*-axis refers to the −log10 of FDR achieved by LinDA. The untransformed FDRs are displayed in parentheses. (C, D) Venn diagrams showing the overlap number of species in subgingival plaques tested by the two methods with FDR < 0.1 (**C**) and FDR < 0.05 (**D**). (E) Identification of salivary bridge species. A total of 127 species (selected by mean relative abundance >0.001) in saliva were tested the same as panel **B**. 28 salivary bridge species were identified, including 25 depleted and 3 enriched in PDHTN. (F, G) Venn diagrams showing the overlap number of species in saliva tested by the two methods with FDR < 0.1 (**F**) and FDR < 0.05 (**G**).

**Fig 3:**
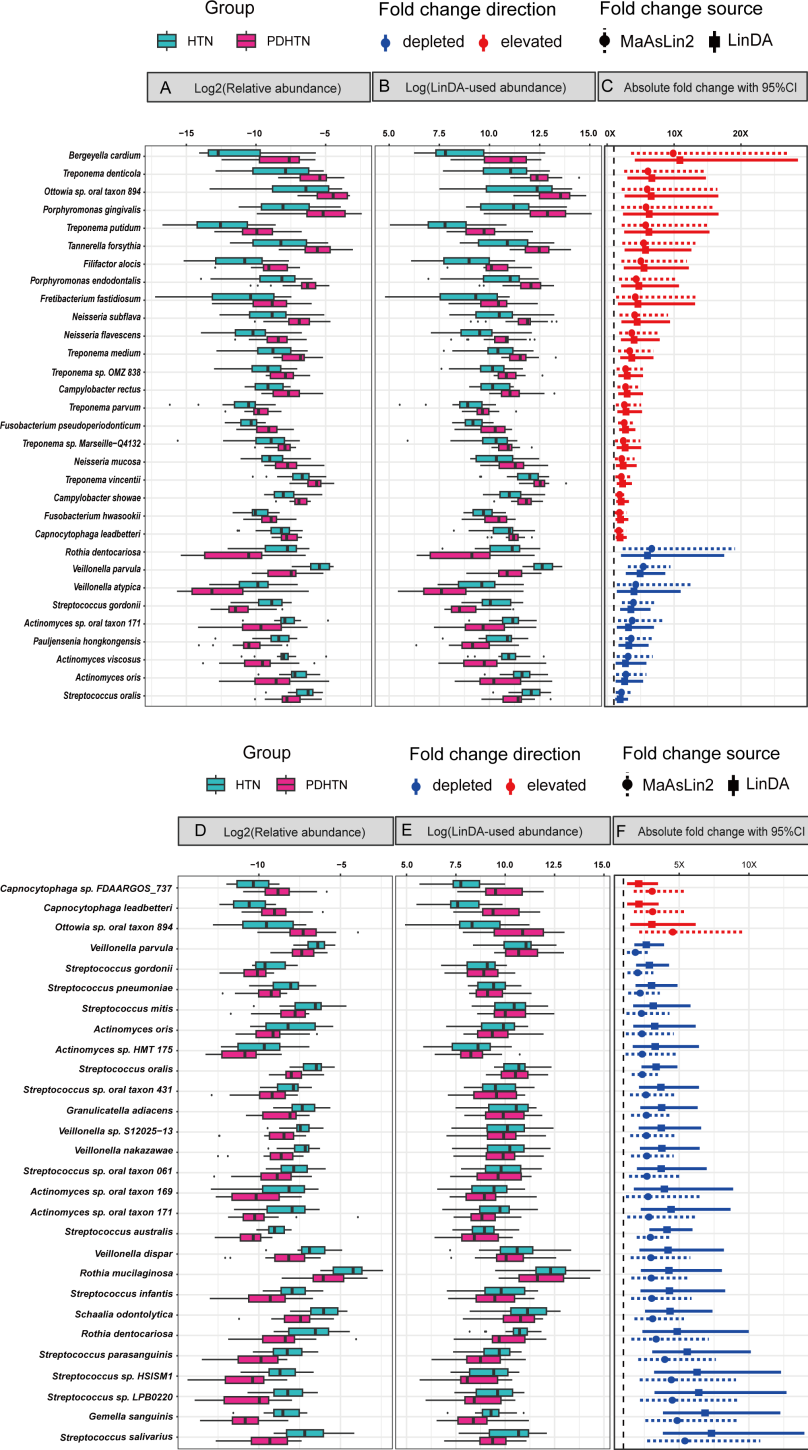
Differential abundances and fold changes of bridge species. (A) Boxplots showing the log2-transformed relative abundance values of subgingival bridge species used in MaAsLin2. (B) Boxplots showing natural log-transformed LinDA-modified abundance values of subgingival bridge species. (C) Absolute fold changes of subgingival bridge species in HTN vs PDHTN. (D) Boxplots showing the log2-transformed relative abundance values of salivary bridge species used in MaAsLin2. (E) Boxplots showing natural log-transformed LinDA-modified abundance values of salivary bridge species. (F) Absolute fold changes of salivary bridge species in HTN vs PDHTN.

Next, to explore what microbial community under periodontitis state plays an important role in exacerbating hypertension, we analyzed the differentially distributed microbial species between HTN and PDHTN in subgingival plaques and saliva, via MaAsLin2 and LinDA (Data S1 and S2). A species was identified as a bridge species (species serving as pivotal links in the exacerbation of hypertension induced by periodontal disease) if it achieved an FDR (false discovery rate) of <0.05 by one method and <0.1 by the other (Fig. 2A). According to these criteria, 31 subgingival bridge species were defined, 24 of which achieved an FDR < 0.05 by both MaAsLin2 and LinDA, and 7 of which had an FDR < 0.05 according to MaAsLin2 with an FDR < 0.1 according to LinDA (Fig. 2B through D). Among the 31 subgingival bridge species, 22 were enriched and 9 were depleted in PDHTN; specifically, 71% of the species were enriched in PDHTN (Fig. 3A through C, Table 1). 28 salivary bridge species were defined, 23 of which achieved an FDR < 0.05 by both MaAsLin2 and LinDA, and 5 of which had an FDR < 0.05 by Linda and an FDR < 0.1 by MaAsLin2 (Fig. 2E through G). Among the 28 salivary bridge species, 3 were enriched, and 25 were depleted in PDHTN; specifically, 90% of the species were depleted in PDHTN (Fig. 3D through F, Table 2).

**TABLE 1:** Characterization of 31 identified subgingival bridge species^*a*^

Identified species	Prevalence		Maaslin2 results		LinDA results		Cluster#	Analysis of confounding
	HTN	PDHTN	FDR	FC	FDR	FC		
*Treponema denticola*	16	19	0.0063655	6.0935725	0.0014724	6.6775048	1	+
*Porphyromonas gingivalis*	16	19	0.0134533	5.8039396	0.0058914	6.2830656	1	+
*Treponema putidum*	16	19	0.0127433	5.7520759	0.0035007	6.2361779	1	+
*Tannerella forsythia*	16	19	0.0117171	5.4593942	0.0027633	5.7314298	1	+
*Filifactor alocis*	16	19	0.0117171	5.0355943	0.0035007	5.5084355	1	+
*Porphyromonas endodontalis*	16	19	0.0182954	4.3258326	0.0058914	4.7284749	1	/
*Fretibacterium fastidiosum*	16	19	0.0678268	4.2025101	0.0315009	4.5976467	1	/
*Treponema medium*	16	19	0.0160066	3.3706041	0.0035007	3.6747578	1	/
*Treponema* sp. OMZ 838	16	19	0.029986	2.7529938	0.0058914	2.9978044	1	/
*Campylobacter rectus*	16	19	0.013297	2.7463252	0.0061128	2.9841362	1	+
*Treponema parvum*	16	19	0.0486365	2.5683089	0.0128185	2.8118593	1	/
*Fusobacterium pseudoperiodonticum*	16	19	0.0063655	2.5500437	0.0014724	2.7453541	1	+
*Treponema* sp. Marseille-Q4132	16	19	0.0702925	2.4085055	0.0233708	2.6863169	1	/
*Treponema vincentii*	16	19	0.0524377	2.1152684	0.0095813	2.3014763	1	/
*Fusobacterium hwasookii*	16	19	0.0647227	1.8319031	0.0265196	1.9956652	1	/
*Rothia dentocariosa*	16	19	0.0127433	0.1502596	0.0128185	0.1660627	1	−
*Actinomyces* sp. oral taxon 171	16	19	0.0232523	0.2676046	0.0307186	0.3147339	1	−
*Actinomyces viscosus*	16	19	0.0380564	0.3198392	0.0493664	0.3657331	1	−
*Actinomyces oris*	16	19	0.0486365	0.3575731	0.0490948	0.3837998	1	−
*Streptococcus oralis*	16	19	0.0342481	0.4687719	0.0419404	0.5172642	1	/
*Bergeyella cardium*	16	19	0.0038266	9.8681373	0.0014724	10.866584	2	+
*Campylobacter showae*	16	19	0.0401917	1.8608703	0.0128185	2.0945305	2	/
*Veillonella parvula*	16	19	0.0001349	0.1849322	0.0004789	0.2032583	2	−
*Veillonella atypica*	16	19	0.0530317	0.2342906	0.0419772	0.2501017	2	/
*Pauljensenia hongkongensis*	16	19	0.0117171	0.279152	0.0082574	0.3096539	2	−
*Ottowia* sp. oral taxon 894	16	19	0.0127433	5.9921305	0.0035007	6.574727	3	+
*Neisseria subflava*	16	19	0.0127433	4.1070794	0.0035007	4.4781131	3	+
*Neisseria flavescens*	16	19	0.0127433	3.6730616	0.0035007	4.0233284	3	+
*Neisseria mucosa*	16	19	0.0702925	2.1746409	0.0352361	2.3864395	3	/
*Capnocytophaga leadbetteri*	16	19	0.0976111	1.7452869	0.0193941	1.9291675	4	/
*Streptococcus gordonii*	16	19	0.0031436	0.2547207	0.0035007	0.282241	4	−

^*a*^ Prevalence refers to the detection frequency of a species among individuals. HTN, participants with hypertension; PDHTN, participants with periodontitis and hypertension; FDR, false discovery rate; FC, fold change; Cluster# refers to the corresponding numbers in Fig. 4A. For analysis of confounding, + refers to the species that remained to be associated with HTN-aggravating effect of PD (bridge species) after adjusting for three variables (age, gender, and BMI) and were enriched in PDHTN; − refers to the species that remained to be associated with HTN-aggravating effect of PD (bridge species) after adjusting for three variables and were depleted in PDHTN; / refers to the species that lost association with the HTN-aggravating effect of PD (not bridge species).

**TABLE 2:** Characterization of 28 identified salivary bridge species^*a*^

Identified species	Prevalence		Maaslin2 results		LinDA results		Cluster#	Analysis of confounding
	HTN	PDHTN	FDR	FC	FDR	FC		
*Rothia dentocariosa*	16	19	0.0207471	0.2987157	0.0009504	0.2058648	1	/
*Actinomyces* sp. oral taxon 171	16	19	0.0639229	0.3524234	0.0009504	0.2263352	1	/
*Actinomyces* sp. oral taxon 169	16	19	0.0879786	0.3610051	0.0106901	0.2540114	1	/
*Actinomyces* sp. HMT 175	16	19	0.0879786	0.4260557	0.0078057	0.305026	1	/
*Actinomyces oris*	16	19	0.081637	0.4292166	0.0059556	0.3080085	1	/
*Streptococcus australis*	16	19	0.0012358	0.3403287	1.00E−06	0.2423451	2	−
*Ottowia* sp. oral taxon 894	16	19	0.0050441	4.537605	0.0183127	3.0374681	3	+
*Capnocytophaga leadbetteri*	16	19	0.0050441	3.0928489	0.0273907	2.1167394	3	+
*Capnocytophaga* sp. FDAARGO737	16	19	0.0050441	3.0620709	0.0275604	2.096728	3	+
*Streptococcus salivarius*	16	19	0.0014839	0.1848258	2.80E−05	0.1366714	3	−
*Streptococcus* sp. LPB0220	16	19	0.004409	0.2219589	0.0001147	0.1561737	3	−
*Streptococcus* sp. HSISM1	16	19	0.004409	0.225188	0.0001147	0.159227	3	−
*Streptococcus parasanguinis*	16	19	0.004409	0.2521049	7.10E−05	0.1792881	3	−
*Schaalia odontolytica*	16	19	0.004409	0.3229108	0.0001113	0.2302316	3	−
*Veillonella dispar*	16	19	0.0206503	0.3365101	0.0012023	0.2380721	3	−
*Veillonella nakazawae*	16	19	0.0110831	0.3737825	0.0003735	0.2661376	3	−
*Veillonella s*p. S12025-13	16	19	0.0172715	0.3777993	0.0006465	0.2689848	3	−
*Streptococcus gordonii*	16	19	0.0215209	0.4951366	0.00017	0.3492816	3	/
*Veillonella parvula*	16	19	0.0207471	0.5376137	0.0003317	0.3784187	3	/
*Gemella sanguinis*	16	19	0.0014839	0.2060477	1.40E−05	0.1458933	4	−
*Streptococcus infantis*	16	19	0.0173614	0.3300045	0.0009233	0.2325602	4	/
*Rothia mucilaginosa*	16	19	0.0172715	0.3332984	0.0007002	0.2344899	4	−
*Streptococcus* sp. oral taxon 061	16	19	0.0250503	0.371808	0.0016581	0.2696413	4	/
*Granulicatella adiacens*	16	19	0.0110831	0.3781336	0.0002846	0.2670667	4	−
*Streptococcus* sp. oral taxon 431	16	19	0.0173614	0.38273	0.0005523	0.2711036	4	/
*Streptococcus oralis*	16	19	0.0039786	0.4247534	1.70E−05	0.2988437	4	−
*Streptococcus mitis*	16	19	0.0607468	0.435403	0.005154	0.3182943	4	/
*Streptococcus pneumoniae*	16	19	0.0207471	0.454107	0.0006465	0.3310299	4	−

^*a*^ Prevalence refers to the detection frequency of a species among individuals. HTN, participants with hypertension; PDHTN, participants with periodontitis and hypertension; FDR, false discovery rate; FC, fold change; Cluster# refers to the corresponding numbers in Fig. 4B. For analysis of confounding, + refers to the species that remained to be associated with HTN-aggravating effect of PD (bridge species) after adjusting for three variables (age, gender, and BMI) and were enriched in PDHTN; − refers to the species that remained to be associated with HTN-aggravating effect of PD (bridge species) after adjusting for three variables and were depleted in PDHTN; / refers to the species that lost association with the HTN-aggravating effect of PD (not bridge species).

For effect sizes (Fig. 3; Tables 1 and 2), at one extreme, *Bergeyella cardium* showed a 10-fold increase, *Treponema denticola* and *Ottowia* sp. oral taxon 894 exhibited a sixfold increase in subgingival plaques, while *Ottowia* sp. oral taxon 894 was elevated by 4.5-fold in saliva. Conversely, *Rothia dentocariosa* was reduced by 6.5-fold, *Veillonella parvula* by 5.5-fold in subgingival plaques, and *Streptococcus salivarius* by 5.5-fold in saliva. Overall, 87% (27 of 31) of subgingival and all salivary bridge species presented more than twofold changes in abundance according to both statistical methods.

The bridge species that overlapped in saliva and subgingival plaques were *Ottowia* sp. oral taxon 894 and *Capnocytophaga leadbetteri*, whose abundances increased in PDHTN, as well as *Rothia dentocariosa*, *Streptococcus gordonii*, *Streptococcus oralis*, *Veillonella parvula, Veillonella dispar*, *Actinomyces s*p. oral taxon 171, and *Actinomyces oris*, whose abundances decreased in PDHTN.

We also analyzed the differential abundance between HC and PD or HTN in the two sample types (Fig. S3 to S7) and found that five subgingival species showed a differential abundance in the PD group compared to HC (Fig. S1; Table S3), with no significantly distinct microorganisms between HC and HTN in subgingival plaques. Interestingly, 2 PD-associated species, namely, *Fretibacterium fastidiosum* and *Filifactor alocis,* were elevated in PD or HTN when compared to HC in salivary samples (Table S5 and S6). Moreover, 17 of 28 salivary bridge species also presented a significant difference between HC and PD in salivary samples (Fig. S1; Table S5).

To eliminate the effects of potential confounders (16) (i.e., age, gender, and BMI), even though there was no significant statistical difference (Table S1), we retested the bridge species in the MaAsLin2 model including age, gender, and BMI. 18 of 31 subgingival bridge species retained significance at FDR < 0.1 (Table 1) and 17 of 28 salivary bridge species retained significance (Table 2).

### Network analyses to reveal a polymicrobial cluster

**Fig 4:**
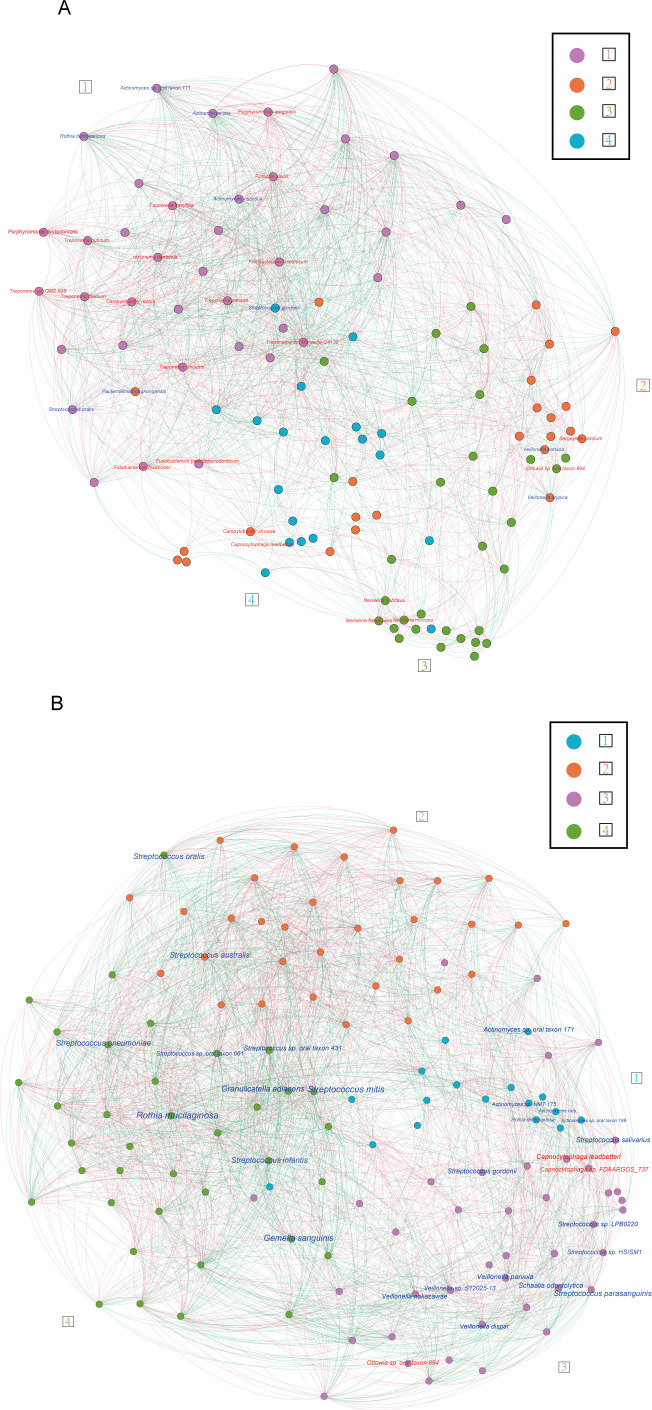
Polymicrobial clusters of co-correlated species in the subgingival plaques and saliva of participants with periodontitis and hypertension. (A, B) Network analysis of the species in the subgingival (**A**) and salivary (**B**) microbiota of participants with PDHTN. Species selected by mean relative abundance >0.001 were calculated for co-correlated analysis using SparCC correlations and used to construct a network if the |*r*| > 0.2 and *P*-value < 0.05. The Louvain algorithm was used to define the clusters, and all clusters were randomly assigned a number and a color. Bridge species were marked into the network and colored in blue if decreased in PDHTN or red if increased in PDHTN. *N* = 19 for subgingival plaques and saliva.

A network of correlated species may reveal polymicrobial clusters related to disease. We calculated pairwise correlations (r) for metagenomes of PDHTN (Data S4 and S6) and HTN (Data S3 and S5) from 113 species in subgingival plaques or 127 species in saliva via two statistical tools. Both positive and negative correlations were observed, including those for bridge species. We employed pairwise correlations with an absolute value greater than 0.2 and permutation *P*-values less than 0.05 to construct correlation networks, identifying clusters of species exhibiting significant correlations via the Louvain algorithm (Fig. 4**,** Fig. S1; Data S7 to S10). We subsequently assigned bridge species to the established networks.

The subgingival bridge species elevated in PDHTN, such as *Tannerella forsythia*, *Treponema denticola*, *Porphyromonas gingivalis*, *Filifactor alocis*, and several other *Treponema* and *Porphyromonas* genera, presented positive correlations with each other (Fig. 4A; Table 1; Data S8). These species were subsequently assigned to cluster #1 within the subgingival PDHTN network. Within Cluster #1, there were a total of 39 species, 20 of which were subgingival bridge species. Among the 20 bridge species, 15 showed an enrichment in abundance in PDHTN, and 5 displayed a reduction. All these species were interconnected through a combination of positive and negative correlations. The remaining species in cluster #1 included 19 species, 2 of which, namely *Fusobacterium nucleatum* and *Prevotella intermedia*, are commonly associated with PD.

Of the subgingival bridge species, the one that showed the greatest decrease was *Rothia dentocariosa*, which was mapped to cluster #1 in the PDHTN network (Fig. 4A; Table 1; Data S8). Moreover, *R. dentocariosa* was negatively correlated with subgingival bridge species increased in PDHTN within cluster #1, suggesting that a competitive relationship occurred in the PDHTN microbiota.

Many depleted salivary bridge species, including *Streptococcus salivarius, Streptococcus gordonii*, and *Veillonella* dispar, were assigned to cluster #3 in the salivary PDHTN network (Fig. 4B; Table 2; Data S10).

The only three enriched bridge species, *Ottowia* sp. oral taxon 894, *Capnocytophaga* sp. FDAARGOS_737, and *Capnocytophaga leadbetteri*, were assigned to cluster #3 in the salivary PDHTN network (Fig. 4B, Table 2; Data S10). Within cluster #3, there were a total of 41 species, 13 of which were salivary bridge species. Among the 13 bridge species, 3 presented an enrichment in abundance in PDHTN, and 10 exhibited a reduction. The remaining species of cluster #3 in the salivary PDHTN network included most of the subgingival bridge species, such as *Bergeyella cardium*, *Campylobacter rectus*, *Campylobacter showae*, *Fusobacterium hwasookii*, and *Porphyromonas endodontalis*, other PD-associated species, including *Prevotella intermedia* and *Capnocytophaga gingivalis*, and another oral pathogen, *Prevotella melaninogenica*.

### *Filifactor alocis* aggravates angiotensin II-induced hypertension in LIP mice

**Fig 5:**
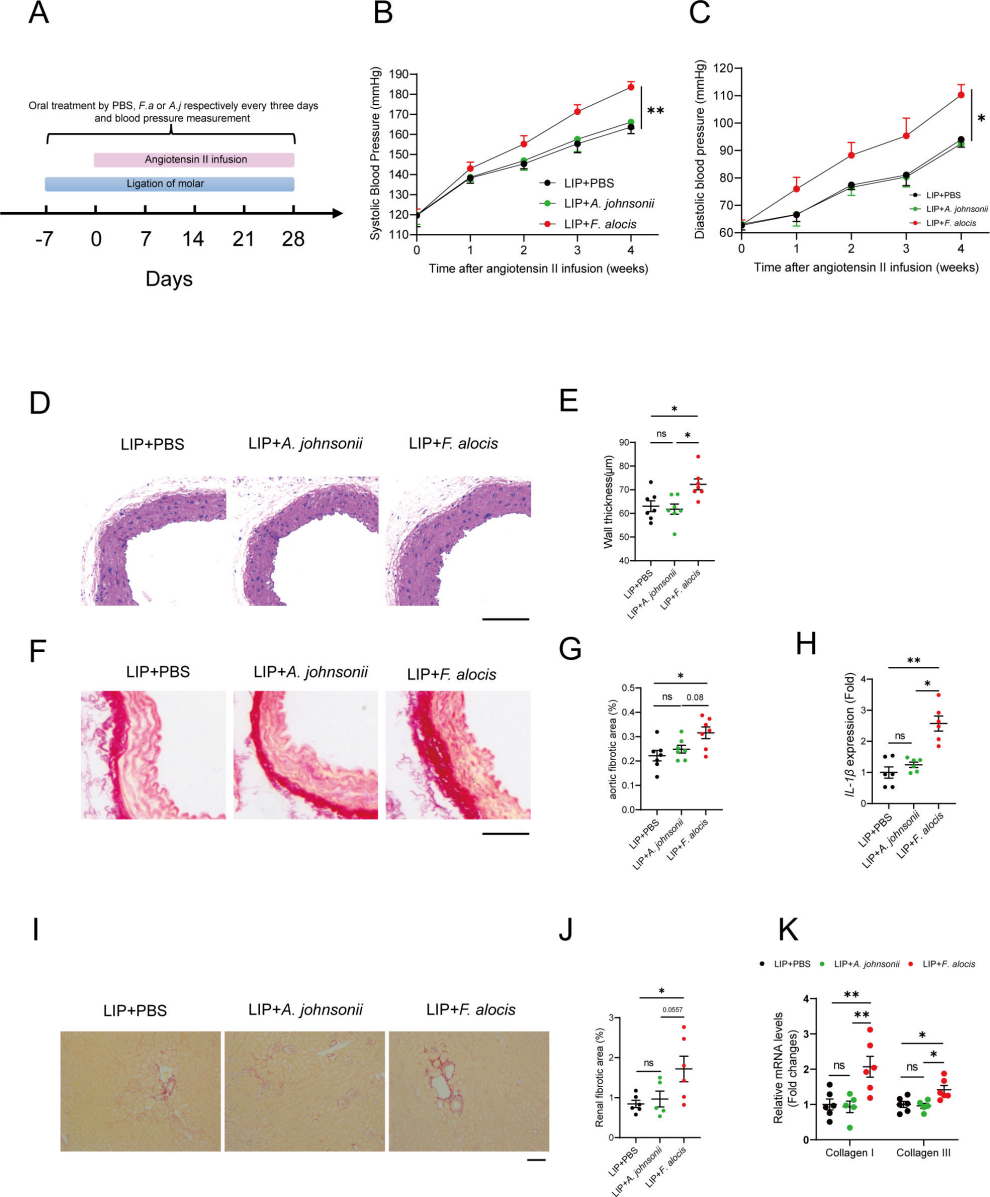
*Filifactor alocis* exacerbates angiotensin II-induced HTN in LIP mice. (A) Scheme of the experimental design. Mice were treated by ligation of the molar at day −7 and were orally swabbed with PBS, *Actinomyces johnsonii* (*A. johnsonii*) or *Filifactor alocis* (*F. alocis*) every 3 days from day −7 to day 28. Hypertensive mice were treated with angiotensin II (Ang II) via subcutaneous infusion using an osmotic pump at day 0 for 4 weeks. (B, C) Noninvasive tail-cuff monitoring of systolic (**B**) and diastolic blood pressure (**C**) of LIP mice treated with PBS, *A. johnsonii,* or *F. alocis* before and after angiotensin II infusion. (D) Representative H&E staining of the thoracic aortas. Scale bar, 100 µm. (E) Quantification of the aortic wall thickness. (F) Representative picrosirius red staining of the thoracic aortas. Scale bar, 100 µm. (G) Quantification of aortic fibrotic areas. (H) qRT-PCR analysis of IL-1β in aortic tissues. (I) Representative picrosirius red staining of the kidneys. (J) Quantification of renal fibrotic areas. (K) qRT-PCR analysis of fibrotic genes in kidneys. Scale bars, 100 µm. LIP, ligature-induced periodontitis. Data were presented as mean ± SEM and analyzed using one-way ANOVA and two-way ANOVA. *N* = 6:6:6 for blood pressure measurement, *N* = 7:7:7 for aortas and *N* = 6:5:6 for kidneys. *adjusted *P* < 0.05, ** adjusted *P* < 0.01.

*Filifactor alocis*, one of the identified subgingival bridge species with a fivefold elevation of abundance in PDHTN, is also a newly appreciated gram-positive periodontal pathobiont (17). The other subgingival bridge species that are elevated in PDHTN are mainly gram-negative pathogens; thus, we selected *F. alocis* to investigate whether it could aggravate hypertension in mice. *Actinomyces johnsonii* (*A. johnsonii*), a gram-positive bacterium isolated from subgingival plaques in PDHTN, was used as a negative control (Fig. 5A). Oral administration of *F. alocis* or *A. johnsonii* exacerbated resorption of alveolar bone (Fig. S3A and B) and increased inflammation in the gingival tissues of LIP mice (Fig. S3C). However, *F. alocis,* but not *A. johnsonii,* significantly elevated both SBP and DBP compared with PBS in hypertensive LIP mice (Fig. 5B and C).

Vascular damage is an important manifestation of hypertension. The results showed that aortic wall thickness (Fig. 5D and E) and collagen deposition (Fig. 5F and G) were strikingly elevated in *F. alocis*-treated mice compared with those in PBS- or *A. johnsonii*-treated mice. The expression of interleukin-1β (IL1β) was strikingly increased in the aortic tissues of *F. alocis*-treated mice (Fig. 5H). High blood pressure also causes renal damage. The manifestations of renal fibrosis in *F. alocis*-treated mice were significantly worse than the other two groups (Fig. 5I through K). All these data showed that *F. alocis* aggravates angiotensin II-induced hypertension in LIP mice.

### *Filifactor alocis* promotes renal infiltration of IFNγ^+^ T cells in angiotensin II-induced hypertension in LIP mice

**Fig 6:**
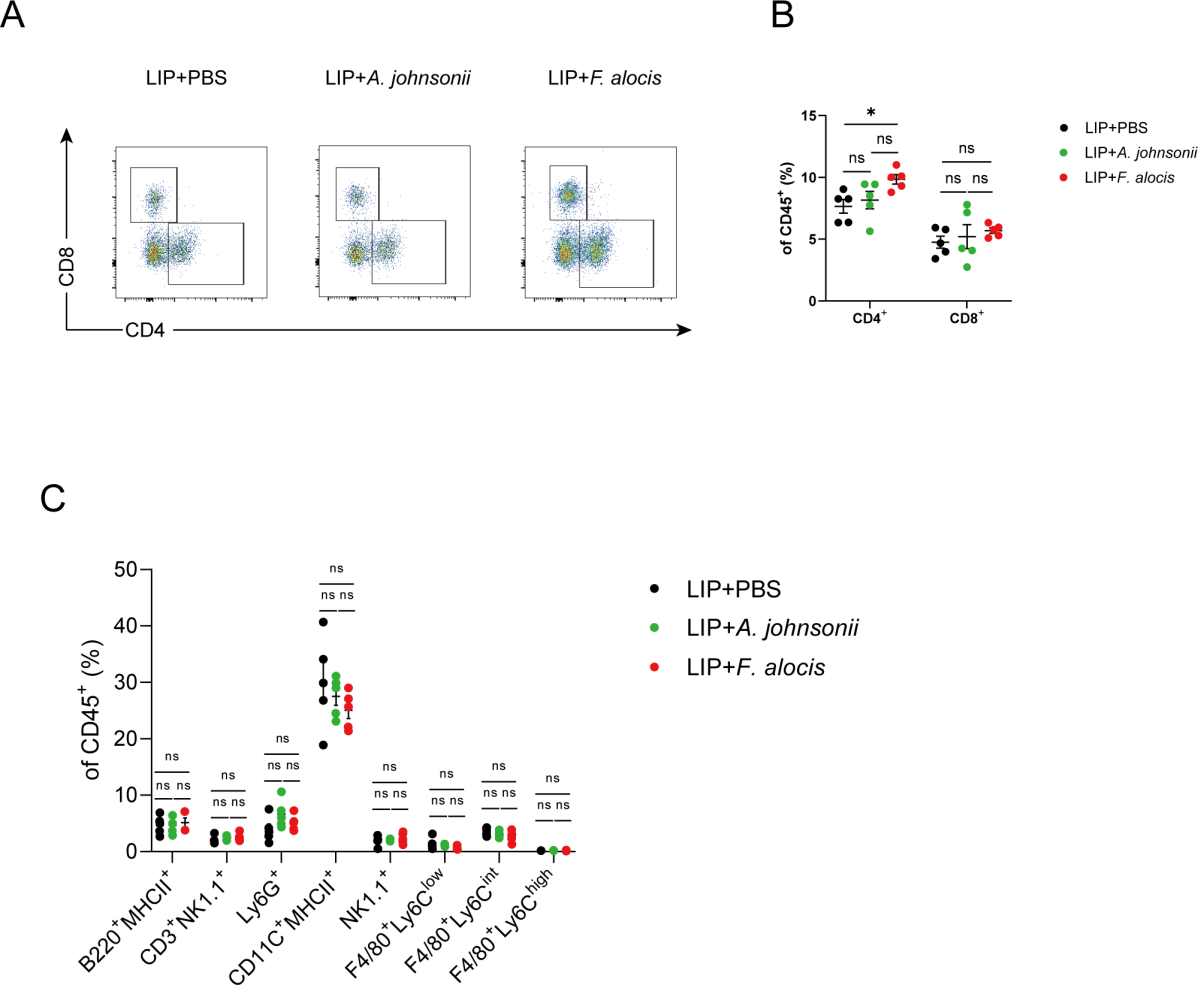
*Filifactor alocis* increases CD4^+^ T-cell infiltration in kidneys of angiotensin II-infused LIP mice. (A) Representative flow cytometry analysis of T cells in the mice kidneys. (B) Quantification of the percentage of CD4^+^ T cells and CD8^+^ T cells in CD45^+^ cells in the mice kidneys. (C) Quantification of the percentage of lymphoid and myeloid cells in CD45^+^ cells in the mice kidneys. LIP, ligature-induced periodontitis. Data were presented as mean ± SEM and analyzed using one-way ANOVA. *N* = 5:5:5 in each group. * adjusted *P* < 0.05.

Since the immune system plays an important role in the onset and progression of hypertension, and microorganisms can induce an immune response, we focused on the composition of immune cells in the kidney, a target organ of hypertension. The results revealed that there was no significant difference in the composition of immune cells such as cytotoxic T cells (CD8^+^), B cells (B220^+^MHCII^+^), NK T cells (CD3^+^NK1.1^+^), DCs (CD11C^+^MHCII^+^), monocytes/macrophages (F4/80^+^Ly6C^high^, F4/80^+^Ly6C^int^, F4/80^+^Ly6C^low^), and NK (NK1.1^+^) cells among the different groups (Fig. 6A through C). By contrast, the frequency of T helper (CD4^+^) cells significantly increased in the kidneys of *F. alocis*-treated mice (Fig. 6A through C). However, there was no difference in the frequency of T cells in the aortas of these mice (Fig. S7A and B).

**Fig 7:**
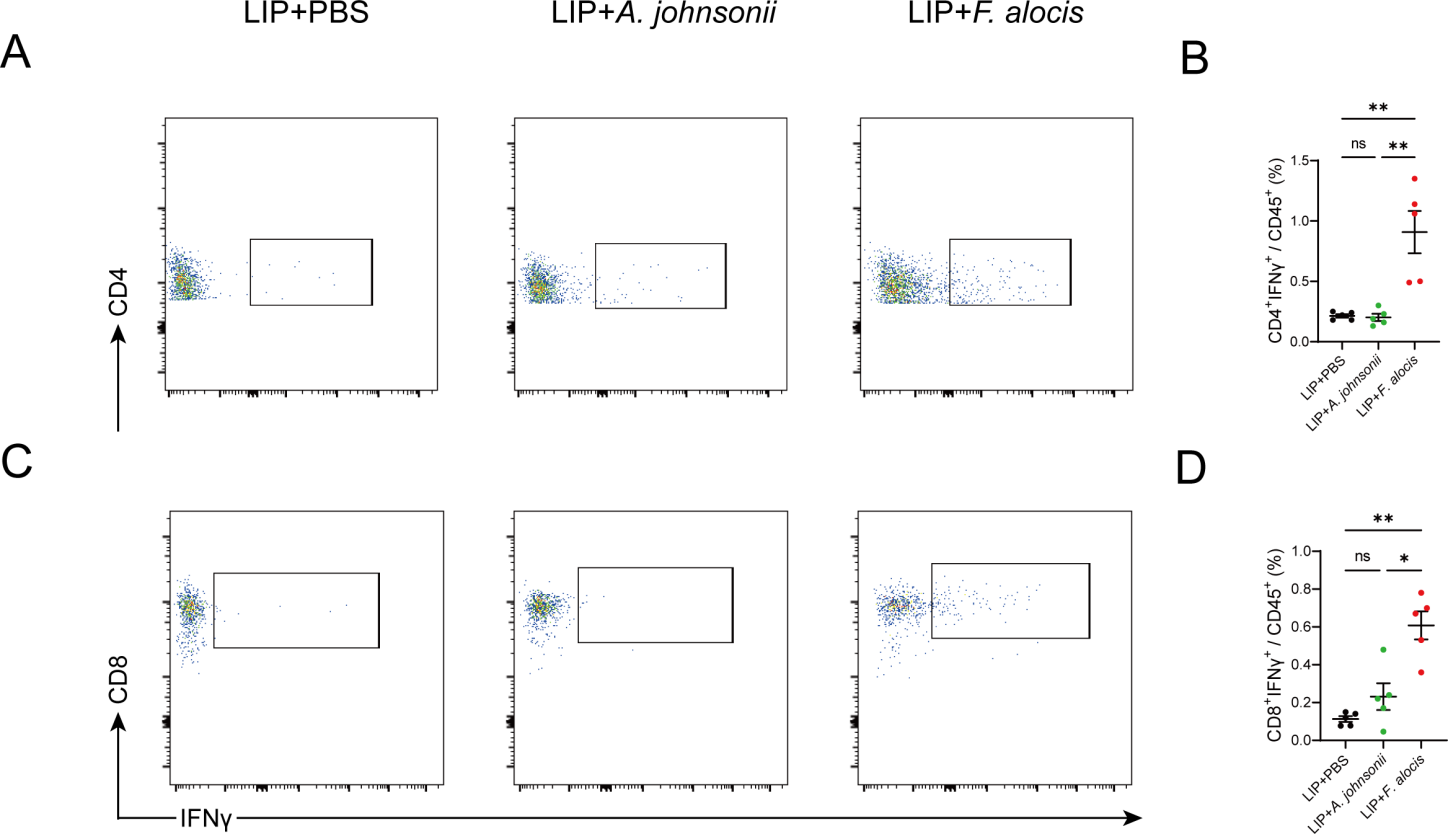
*Filifactor alocis* increases CD4^+^IFNγ^+^ and CD8^+^IFNγ^+^ T-cell infiltration in kidneys of angiotensin II-infused LIP mice. (A) Representative flow cytometry analysis of CD4^+^IFNγ^+^ T cells in the mice kidneys. (B) Quantification of the percentage of CD4^+^IFNγ^+^ T cells in CD45^+^ cells in the mice kidneys. (C) Representative flow cytometry analysis of CD8^+^IFNγ^+^ T cells in the mice kidneys. (D) Quantification of the percentage of CD8^+^IFNγ^+^ T cells in CD45^+^ cells in the mice kidneys. LIP, ligature-induced periodontitis. Data were presented as mean ± SEM and analyzed using one-way ANOVA. *N* = 5:5:5 in each group. *Adjusted *P* < 0.05, ** adjusted *P* < 0.01.

Following the elevation of Th cells in the kidneys of *F. alocis*-treated mice, cytokines (18) produced by T cells, including interleukin-17A (IL17A), interferon-gamma (IFNγ), and tumor necrosis factor alpha (TNFα), play crucial roles in hypertension. We then explore the alteration in the percentage of cytokines in the targeted organ. Results of flow cytometry analysis demonstrated that the kidney samples of *F. alocis*-treated mice had significantly more CD4^+^IFNγ^+^ T cells than those of the other two groups, but both CD4^+^IL17A^+^ and CD4^+^TNFα^+^ T cells were comparable among the groups (Fig. 7A and B; Fig. S6A through D). Interestingly, the percentage of CD8^+^IFNγ^+^ T cells was also significantly higher in the kidney samples of *F. alocis*-treated mice (Fig. 7C and D). Moreover, we also assessed the changes in T-cell-derived cytokines in the blood vessels and found no differences (Fig. S7C through J).

## DISCUSSION

In this study, we explored the links between PD and HTN via metagenome sequencing and identified a list of bridge species that had a potential effect on HTN in the presence of PD. Subsequently, the impacts of *F. alocis* on HTN were verified using an animal model.

Many subgingival bridge species that presented a significant increase in PDHTN were strongly associated with PD. Examples included members of the red complex (11, 19) such as *Tannerella forsythia*, *Treponema denticola*, and *Porphyromonas gingivalis*, as well as other species such as *Filifactor alocis*, *Treponema medium*, *Treponema parvum*, *Porphyromonas endodontalis*, *Campylobacter rectus*, *Campylobacter showae*, and *Fretibacterium fastidiosum* (19–22). Among these species, antigens from *P. gingivalis* can aggravate hypertension and impair vascular function via an enhanced Th1-type immune response in angiotensin II-infused mice (15, 23); Pietropaoli defined *C. rectus* as a hypertension-associated oral pathogen through analysis of the associations between blood pressure and serum antibodies to 21 periodontal microorganisms (24).

Within cluster #1 in the subgingival PDHTN network, there were a total of 39 species, 20 of which were subgingival bridge species. The remaining 19 species in cluster #1, including two PD-associated species, namely, *Fusobacterium nucleatum* and *Prevotella intermedia*, are implicated in many conditions, such as rheumatoid arthritis (25), atherosclerosis (26), colorectal cancer (27), and subclinical hypothyroidism (28). The two species may be implicated in HTN via working synergy with subgingival bridge species or by themselves only.

The bridge species deficient in PDHTN of the subgingival plaques and saliva included *Rothia dentocariosa*, *Rothia mucilaginosa*, *Actinomyces viscosus*, *Actinomyces oris*, *Veillonella parvula*, *Veillonella dispar*, *Veillonella atypica,* and *Granulicatella adiacens*. These bacteria, which are linked to nitrate reduction, might exhibit beneficial effects on HTN (29–32). And those nitrate-reducing bacteria also decreased in the PD group in salivary samples, which is consistent with the latest research (33) that revealed strikingly lower composition of nitrate-reducing bacteria in periodontal patients. Interestingly, the relative abundance of *Neisseria*, a known NO_3_^-^-reducing bacterium (32), which might promote vascular health in saliva via supplementation with dietary nitrate (34), increased in abundance in subgingival plaques in PDHTN. However, *Neisseria* spp. has also been considered a pathobiont, as a study (35) has shown that *Neisseria* spp. is associated with poor clinical outcomes of bronchiectasis. A follow-up animal study has shown that *Neisseria subflava* accelerates the breakdown of epithelial integrity and promotes inflammation (35). It is likely that the subgingival *Neisseria* app. exerts a negative impact on hypertension in the presence of periodontitis. As for the specific effect of *Neisseria* spp. in different oral ecological niches on hypertension, further experiments are needed to validate it.

Among the 25 decreased salivary bridge species, 13 belong to the genus *Streptococcus. Streptococci* are the first organisms detected in newborn infants and are considered pioneer species, playing a pivotal role in maintaining oral health through their colonization (36). For example, hydrogen peroxide produced by *S. oralis*, *S. mitis*, and *S. gordonii* can inhibit the growth of the periodontal pathogen *Porphyromonas gingivalis* (36) that may aggravate angiotensin II-induced hypertension, as discussed above. This finding is consistent with the significant negative correlation between *S. oralis, S. mitis, S. gordonii*, and *P. gingivalis* in the PDHTN network in both saliva and subgingival plaques.

Only three salivary bridge species mapped to cluster #3 increased, and two of these were also subgingival bridge species. There were many subgingival bridge species and pathobionts residing in cluster #3 in the salivary PDHTN network, as listed in Data S10, and most of them were elevated in PD when compared to HC in salivary samples. These species, together with salivary bridge species, may collectively aggravate HTN via the oral-gut route (37, 38). Among these species in cluster #3, *Prevotella melaninogenica* has been defined as a hypertension-associated bacterium (24).

*F. alocis* may aggravate hypertension via the accumulation of IFNγ^+^ T cells, at least in part, in the kidneys. Our animal data revealed that *F. alocis* aggravated hypertension and induced renal infiltration of IFNγ^+^ T cells. A few studies (39, 40) have shown that IFNγ deficiency can decrease blood pressure in angiotensin II-infused mice. Sun et al. (41) demonstrated that the mineralocorticoid receptor on T cells regulates IFNγ production and that targeted deletion of this receptor significantly reduces blood pressure in angiotensin II-induced hypertension. In the kidney, IFNγ disrupts pressure natriuresis and is essential for the activation of sodium reabsorption in the distal tubule (39). Another study has illustrated that IFNγ can promote the renal proximal tubule cells to produce higher levels of angiotensinogen in rats, leading to the elevation of blood pressure (42). Thus, our results substantiate the hypothesis that microbes can play a bridge role in the relationship between PD and HTN.

Nowadays, research (8) shows that intensive periodontal therapy can improve periodontal status and reduce BP via removal of subgingival plaque. Despite the implementation of periodontal therapeutic interventions, including scaling and root planning procedures and/or antibiotic regimens, in conjunction with supportive periodontal therapy (SPT), the long-term prevention of recurrent periodontal disease or refractory periodontitis remains clinically challenging due to antibiotic therapy and/or inadequate patient compliance with prescribed SPT protocols (43–45). Given the critical regulatory role of dysbiosis in the microbiota during pathological processes such as periodontitis (11), modulation of the microbiome has emerged as a promising therapeutic strategy (46). Although our study demonstrated the significant biological impact of *F. alocis* on blood pressure homeostasis, the synergistic and competitive mechanisms involving other bridge species remain incompletely elucidated. Future investigations should prioritize delineating the functional characteristics of individual microbes, deciphering interspecies interaction networks, and clarifying host-microbe interaction mechanisms. These efforts will provide a robust theoretical foundation for developing precise microbiome-based interventions.

Although our study revealed a list of species linking PD and HTN, some limitations existed. For example, the sample size was small, and this was a cross-sectional study. Comparable sample size or experimental designs is used in other studies on oral microbiota (47, 48). The restricted sample size reduced statistical power to detect the effect size. For instance, *Prevotella intermedia* (FDR = 0.10791 for MaAsLin2, FDR = 0.04475 for LinDA) might have reached significance with a larger cohort. Simultaneously, a cross-sectional study was not possible to reveal the causation of bridge species on PD and HTN. Hence, large-scale and longitudinal studies are needed to consolidate our findings.

In summary, our study revealed that the oral microbiome acts as a bridge between periodontitis and hypertension, with a decrease in the abundance of a set of health-associated bacteria and an increase in a group of disease-associated bacteria. Animal studies demonstrated that *F. alocis* might aggravate hypertension by promoting infiltration of IFNγ^+^ T cells in the kidney. All these findings can help grasp the microbial connections between PD and HTN and support joint prevention and control of high blood pressure and periodontitis through modulating these bridge species via microbiome-based therapy in the future.

## MATERIALS AND METHODS

### Study cohorts

The clinical metagenome sequence data analyzed in the present study were obtained from our previous study (6), with the exclusion of diabetic patients. In brief, a total of 57 participants (14 HC, 8 PD, 16 HTN, and 19 PDHTN) were included in this cross-sectional study. The corresponding demographics and clinical parameters are shown in Table S1. STORMS checklists can be found at https://github.com/hamotonz/HTN-PDHTN.

### Metagenomic sequencing

Metagenomic sequencing was conducted on an Illumina NovaSeq platform at Personal Biotechnology Co., Ltd. (Shanghai, China). All raw reads were subjected to quality control using fastp (49). To eliminate host-derived contamination, filtered reads were aligned to the human genome, and the resulting clean reads were used to construct the metagenome. Kraken2 (50) was used to annotate the taxonomic information.

### Animal experiments

Male C57BL/6J mice were purchased from Vital River (Beijing, China). The maxillary second molars were ligatured using 5-0 silk thread saturated with bacteria or phosphate-buffered saline (PBS). One week later, minipumps (2004D, Alzert, Cupertino, California) were subcutaneously implanted to deliver angiotensin II (750 ng/kg/min). Blood pressure was measured using a BP-2000 Blood Pressure Analysis System (Visitech Systems, North Carolina).

### Bacterial culture and oral administration

*Filifactor alocis* (*F. alocis*, CCUG 47,790T) was purchased from Culture Collection University of Gothenburg (CCUG; Sweden) and was cultured in brain heart infusion (BHI) agar with sheep serum, arginine, and cysteine, or broth with hemin, vitamin K, arginine, and cysteine at 37℃ under anaerobic conditions. *Actinomyces johnsonii* (*A. johnsonii*) was isolated from human subgingival plaques, and the culture conditions were the same as those used for *F. alocis*.

After the molars were ligatured, the mice were orally inoculated with 10^8^–10^9^ colony-forming units (CFUs) of bacteria suspended in PBS every 3 days until the experiment ended.

### Histological analysis

Thoracic aorta and kidney samples were fixed in 4% paraformaldehyde, embedded in paraffin, and sectioned at 7 µm thickness. The sections were stained with hematoxylin and eosin (H&E) or 0.1% picrosirius red. All images were captured using a Leica DMi8 microscope (Leica, Wetzlar, Germany) and analyzed with ImageJ software (National Institutes of Health, Bethesda, USA).

### Quantitative real-time polymerase chain reaction (qRT-PCR)

Total RNA was extracted using TRIzol reagent (Thermo Fisher Scientific, Massachusetts, USA), and reverse transcription was carried out with the PrimeScript RT reagent Kit (Takara, Shiga, Japan). qRT-PCR was conducted on a LightCycler 480 II (Roche, Basel, Switzerland) with SYBR Green Mix (Takara, Shiga, Japan). The primers are available in Table S2.

### Flow cytometry analysis

Kidneys and aortas were harvested, sliced, and then placed in Hanks’ balanced salt solution containing collagenase II (1.5 mg/mL for kidney, 1 mg/mL for aorta, Worthington), collagenase IV (1.5 mg/mL for kidney, 1 mg/mL for aorta, Worthington), and 60 U/mL DNase I (Applichem). For kidney samples, single-cell suspension was acquired via mechanical dissociation via a gentleMACS Dissociator system (Miltenyi Biotec) and filtration using a 70 µm cell strainer (BD Biosciences). Aorta samples were minced with fine scissors to obtain a single-cell suspension. All the detailed procedures were described previously (51).

The following antibodies were used: Fixable Viability Stain 510 (564406, BD Biosciences), Zombie NIR Fixable Viability Kit (423105, BioLegend), Fc block (553142, BD Biosciences), CD45 (Clone, 30-F11), B220 (Clone, RA3-6B2), CD3e (Clone, 145–2C11), CD4 (Clone, RM4-5), CD8a (Clone, 53–6.7), NK1.1 (Clone, PK136), MHCII (Clone, M5/114.15.2), CD11b (Clone, M1/70), Ly6G (Clone, 1A8), Ly6C (Clone, AL-21), F4/80 (Clone, BM8), CD11c (Clone, N418), IFNγ (Clone, XMG1.2), TNFα (Clone, MP6-XT22), and IL17A (Clone, TC11-18H10.1).

### Statistical analysis

All analyses were processed by R (4.3.2) and GraphPad Prism 9. The chisq.test function was used to test the categorical variables, the aov function was used for continuous variables, and the vegan package (2.6–4) and ape package (5.7–1) were used for diversity analysis. One-way ANOVA and two-way ANOVA were used to compare the data of three experimental groups in GraphPad Prism and Bonferroni for multiple tests correction. Plotting was completed with ggplot2 (3.4.2). Venn diagrams were plotted by the VennDiagram package (1.7.3).

For differential abundance analysis (DAA) (52), two statistical methods, MaAsLin2 (53) and LinDA (54), were used in parallel. The methodology is referred to in reference 55. The combination of the two linear model-based methods can make the DAA more robust. The MaAsLin2 function from MaAsLin2 (1.14.1) and the linda function from MicrobiomeStat (1.1) were used to achieve the two methods. Species were filtered out using a mean relative abundance of <0.001 as the criterion. The two multivariable association methods have robust performance in maintaining low FDR (54, 56). To correct multiple testing in species-level analysis, we applied the Benjamini-Hochberg false discovery rate (FDR) method (57). For determining statistical significance at the species level, bridge species were identified using an FDR threshold of <0.05 in one statistical method and <0.1 in the other. The effect size represented by the fold change (FC) was calculated in R via equation 1 for MaAsLin2 and LinDA as follows:


(1) $$Fold\;change\;=\;2^{\Lambda }x$$

where *x* represents the vector of model coefficients derived from MaAsLin2 and LinDA.

For age, body mass index (BMI), and sex adjustment (analysis of confounding), 31 subgingival and 28 salivary bridge species were retested via MaAsLin2. Because involving the bias correction for compositional effect, LinDA was not used to retest the bridge species.

SparCC correlation network analysis was performed via the SpiecEasi package (1.1.3), and community analysis was performed by the Louvain algorithm from igraph (1.6.0) in R (4.3.2). Visualization of networks was performed by Gephi via SparCC correlations filtered by *P*＜0.05 and |*r*|＞0.2, and cluster numbers generated by the Louvain algorithm.

## Data Availability

All data are shown in the text and supplemental material. Raw sequence data can be accessed in the Sequence Read Archive of NIH (accession numbers PRJNA765566).

## SUPPLEMENTAL MATERIAL

Supplemental figures msystems.00084-25-s0001.pdf Figures S1 to S7.

Supplemental data msystems.00084-25-s0002.xlsx Data S1 to S10.

Supplemental tables msystems.00084-25-s0003.docx Tables S1 to S7.

ASM does not own the copyrights to Supplemental Material that may be linked to, or accessed through, an article. The authors have granted ASM a non-exclusive, world-wide license to publish the Supplemental Material files. Please contact the corresponding author directly for reuse.
