# Supplementary material for: Linking oral microbiota to periodontitis and hypertension unveils that Filifactor alocis aggravates hypertension via infiltration of interferon-γ+ T cells
Source: mSystems. 2025 May 21;10(6):e00084-25. doi: 10.1128/msystems.00084-25 (PMC12172497; doi:10.1128/msystems.00084-25)
Supplement: Supplemental figures — Figures S1 to S7. [file msystems.00084-25-s0001.pdf]

Figure S1. Differential abundance of bridge species in four groups. A, Boxplots showing natural log-transformed LinDA-modified abundance values of subgingival bridge species. **B,** Boxplots showing natural log-transformed LinDA-modified abundance values of salivary bridge species. The red fonts represent the bridge species elevated in PDHTN, whereas the blue fonts depleted in PDHTN. **#** means the bridge species have significant difference between HC and PD.

A

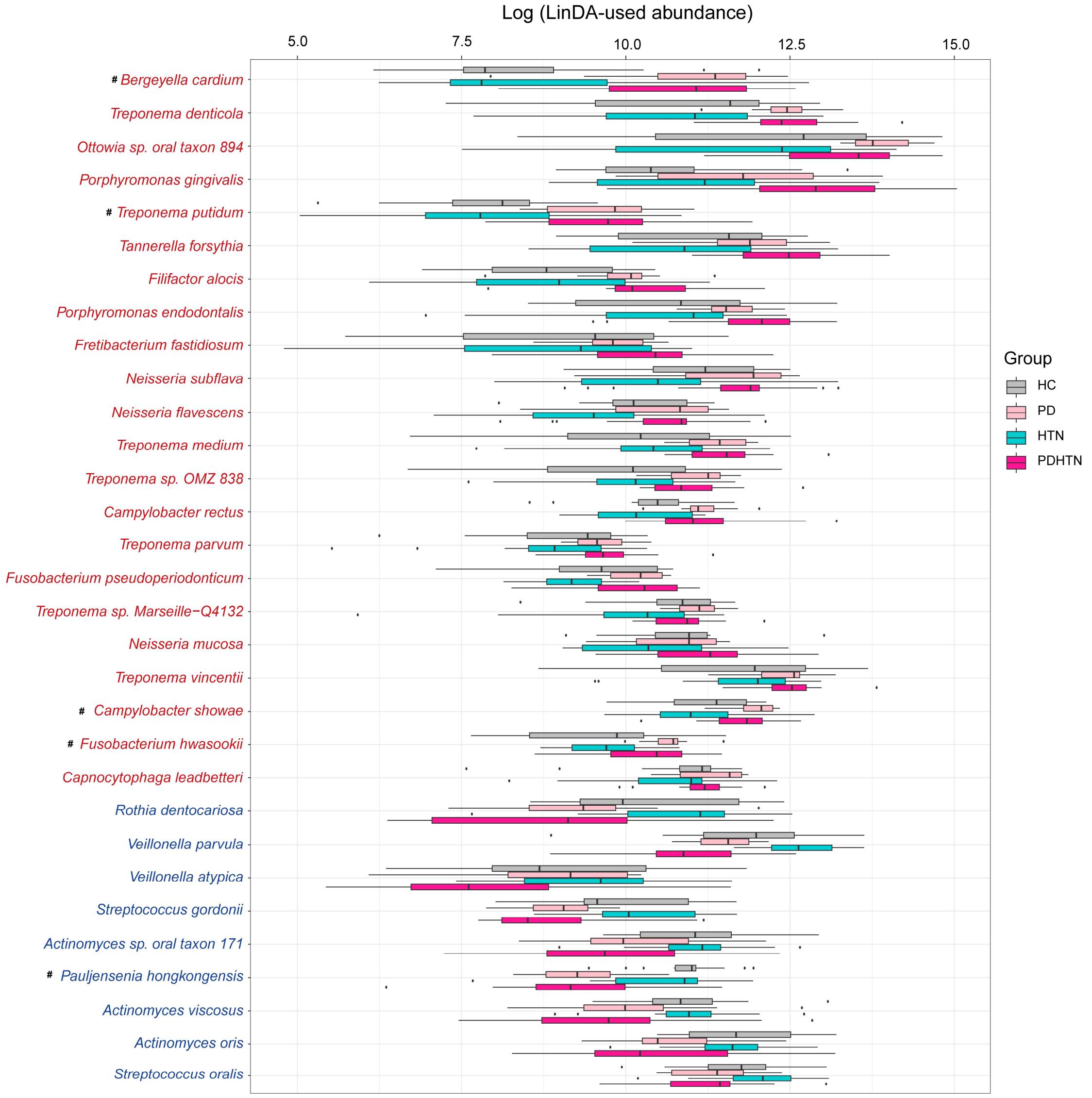

B

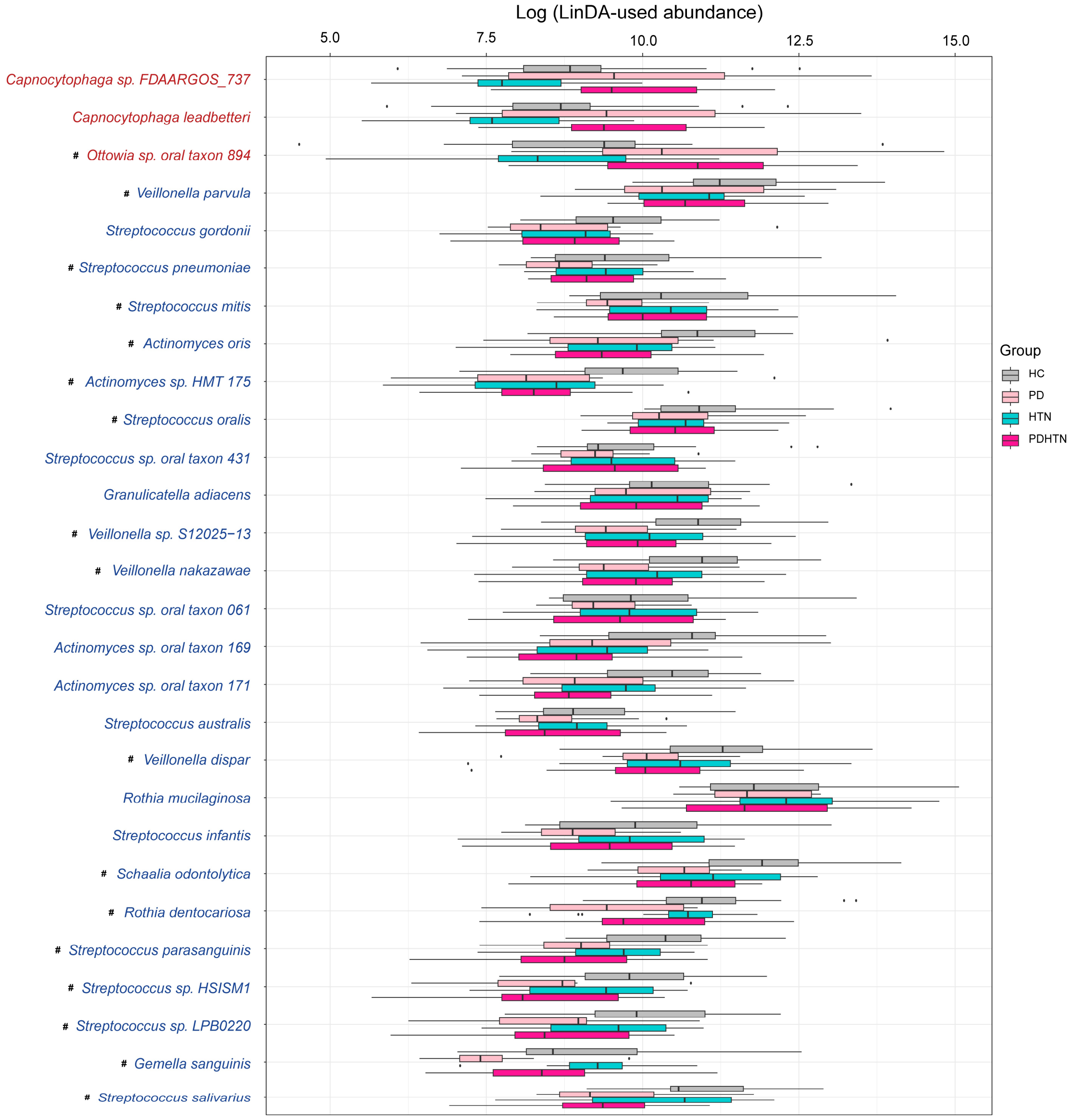

Log (LinDA-used abundance)
5.0
7.5
10.0
12.5
15.0
Capnocytophaga sp. FDAARGOS_737
Capnocytophaga leadbetteri
Ottowia sp. oral taxon 894
Veillonella parvula
Streptococcus gordonii
Streptococcus pneumoniae
Streptococcus mitis
Actinomyces oris
Actinomyces sp. HMT 175
Streptococcus oralis
Streptococcus sp. oral taxon 431
Granulicatella adiacens
Veillonella sp. S12025−13
Veillonella nakazawae
Streptococcus sp. oral taxon 061
Actinomyces sp. oral taxon 169
Actinomyces sp. oral taxon 171
Streptococcus australis
Veillonella dispar
Rothia mucilaginosa
Streptococcus infantis
Schaalia odontolytica
Rothia dentocariosa
Streptococcus parasanguinis
Streptococcus sp. HSISM1
Streptococcus sp. LPB0220
Gemella sanguinis
Streptococcus salivarius
Group
HC
PD
HTN
PDHTN

Figure S2. Polymicrobial clusters of co-correlated species in the subgingival plaques and saliva. A-B, Network analysis of the species in subgingival HTN (**A**) and salivary HTN (**B**). Species selected by mean relative abundance >0.001 were tested for correlation with each other using SparCC correlations and plotted in a network if the |r| > 0.2 and uncorrected permuted *P*-value < 0.05. Clusters were defined by the Louvain algorithm and were randomly assigned a color and a number. Each node denotes a species and the curved lines connect correlated species. Species associated with HTN-aggravating effect of PD were identified via LinDA and MaAsLin2, mapped to the network, and highlighted in blue if reduced in PDHTN, or red if elevated in PDHTN. N = 16 for HTN and N = 19 for PDHTN in subgingival plaques and saliva.

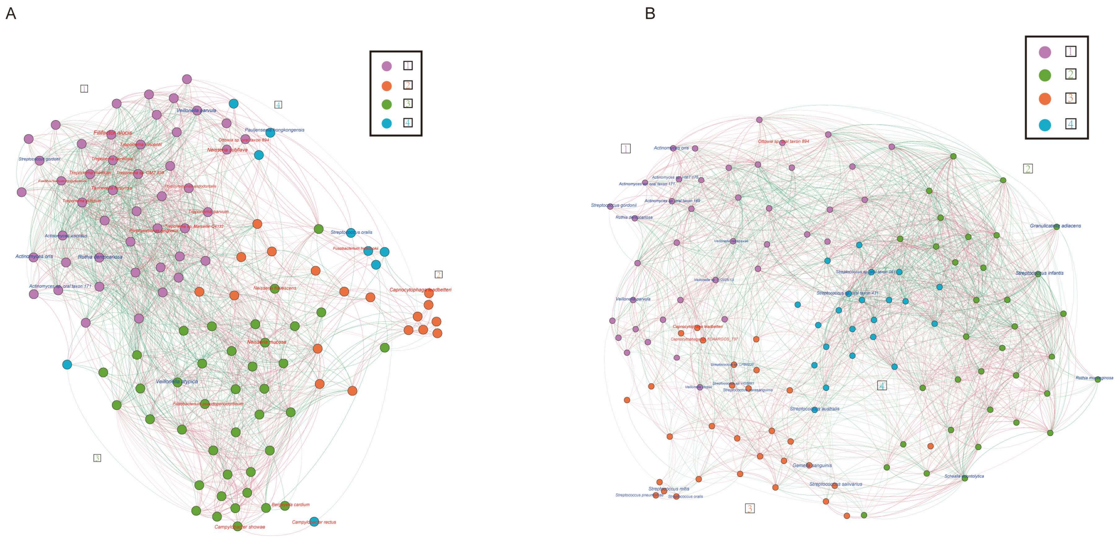

Figure S3. Effects of oral administration by PBS, *A.johnsonii* and *F.alocis* on alveolar bone loss and gingival inflammatory response in angiotensin-II infused and ligated mice.

A, Representative images of maxillary alveolar bone loss. Red solid lines exhibit the space between cementoenamel junction (CEJ) and alveolar bone crest (ABC). Scale bar: 0.5 mm. **B**, CEJ-ABC distance at palatal side of the second molar. n=8:8:10. **C**, qRT-PCR analysis of IL-1β and TNFα in mouse gingival tissues. n=5:5:5 Data are presented as mean ± SEM. One-way ANOVA was used for statistical analysis. *p <0.05; **p <0.01; ***p <0.001; ****p<0.0001.

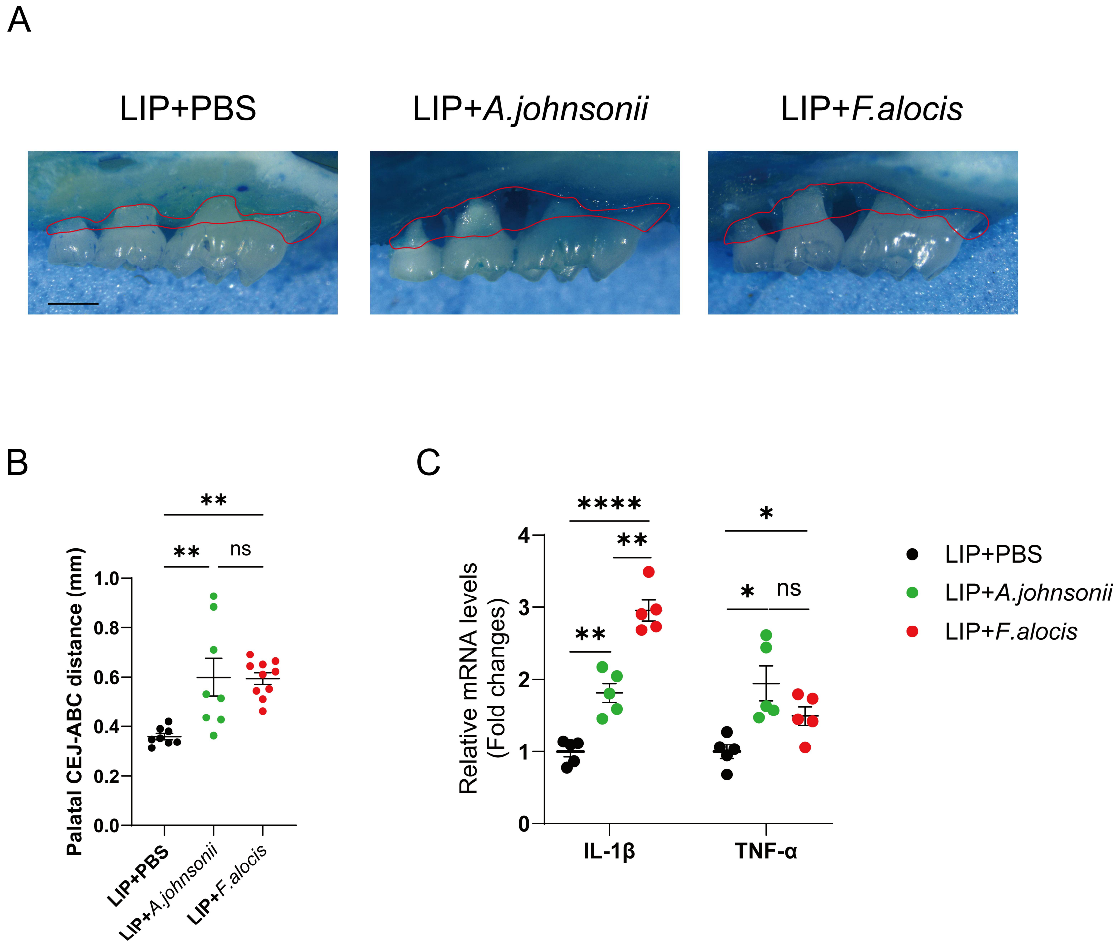

Figure S4. Gate strategy for immune cell analysis in kidney (refer to Figure 6).

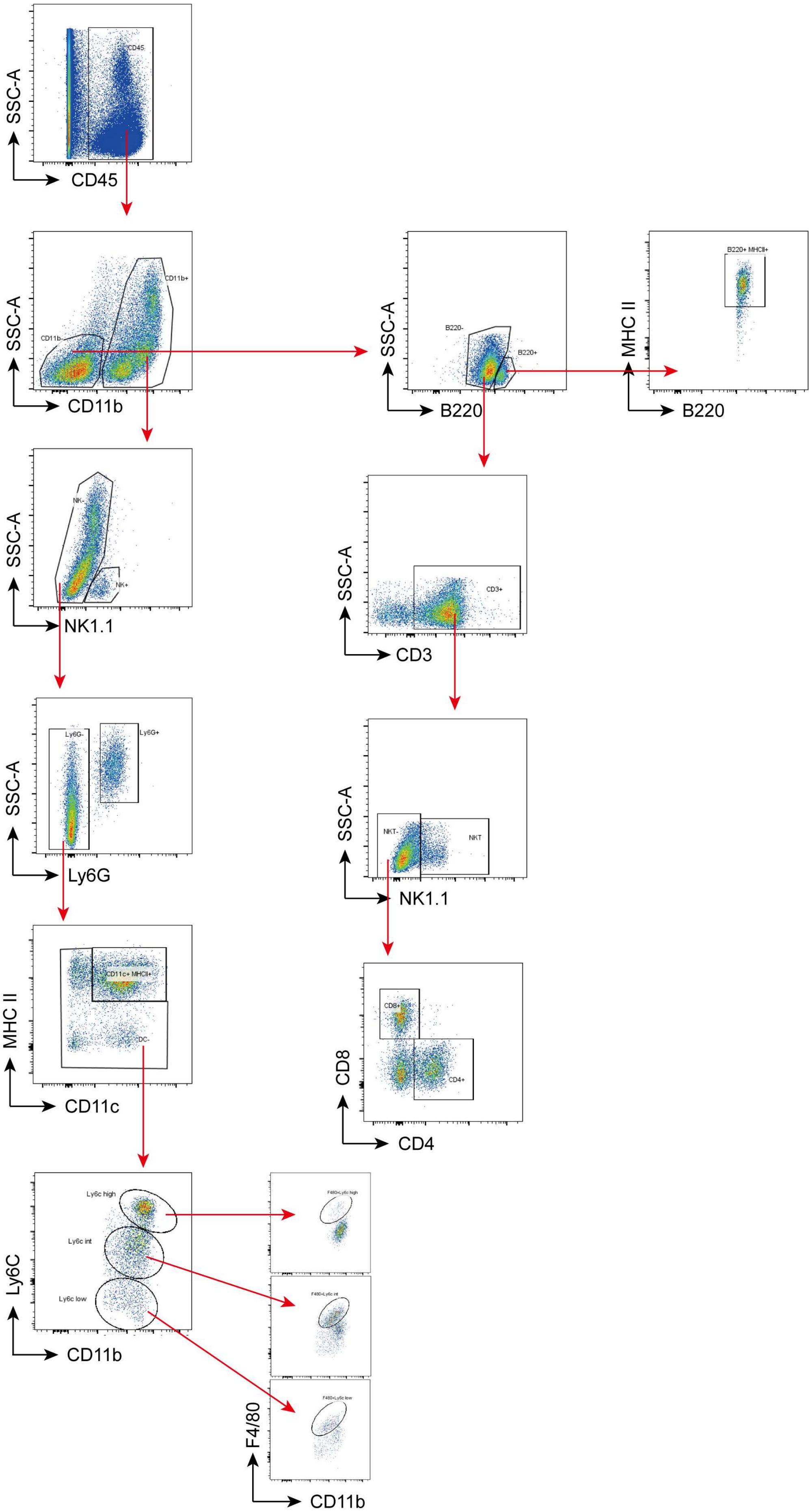

SSC-A
CD45
CD45
CD11b
CD11b-
CD11b+
B220
B220-
B220+
MHC II
B220+ MHCII+
NK1.1
NK-
NK+
CD3
CD3+
Ly6G
Ly6G-
Ly6G+
NKT-
NKT
CD11c
CD11c+ MHCII+
DC-
CD8
CD4
CD8+
CD4+
Ly6C
Ly6c high
Ly6c int
Ly6c low
F4/80
F480+Ly6c high
F480+Ly6c int
F480+Ly6c low

Figure S5. Gate strategy for T cell cytokinesis analysis in kidneys and aortas (refer to Figure 7).

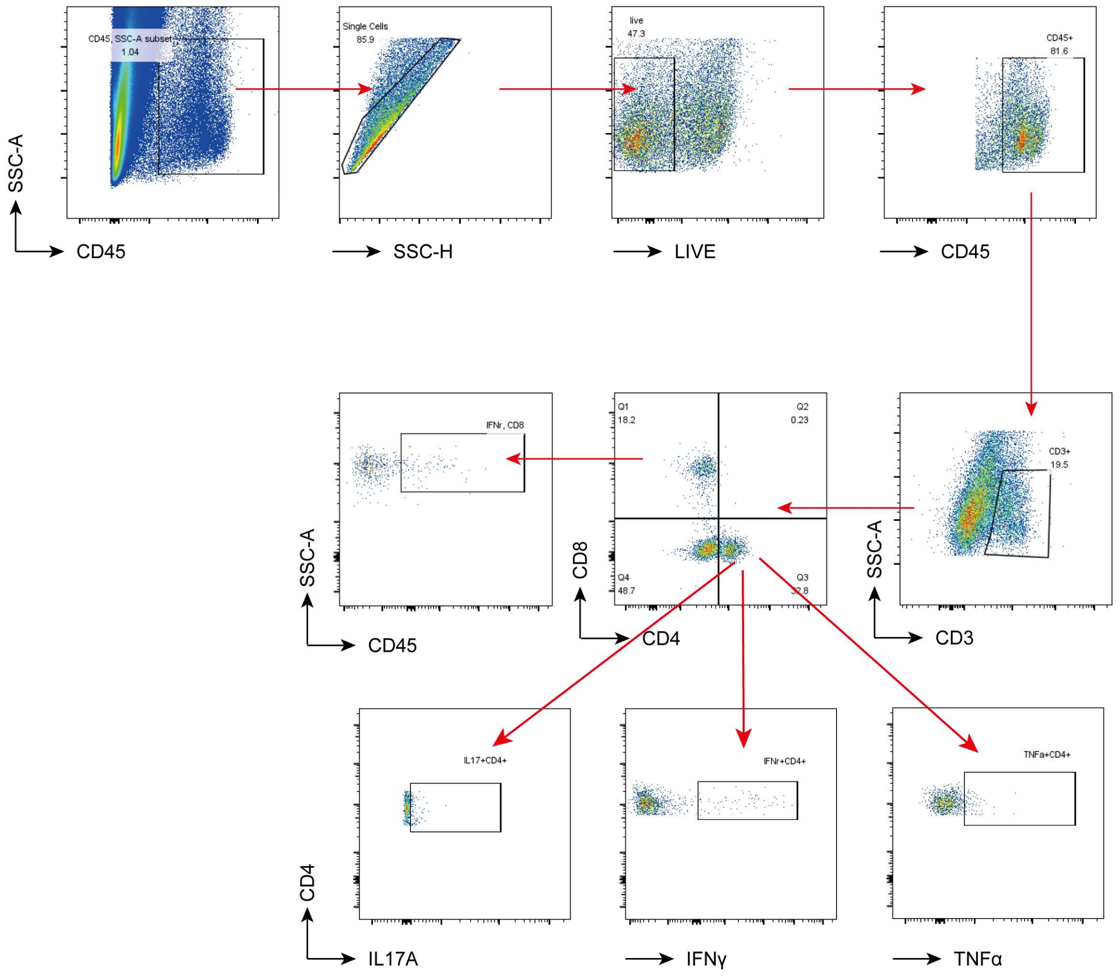

Figure S6. Effect of *Filifactor alocis* on CD4$^+$ IL17A$^+$ and CD4$^+$ TNFα$^+$ T cell infiltration in kidneys of angiotensin ii-infused mice. A-B, Representative flow cytometry image of CD4$^+$ IL17A$^+$ T cell (**A**) and quantification of the percentage of CD4$^+$ IL17A$^+$ T cell in CD45$^+$ cells (**B**) in mouse kidneys. **C-D**, Representative flow cytometry image of CD4$^+$TNFα$^+$ T cell (**C**) and quantification of the percentage of CD4$^+$TNFα$^+$ T cell in CD45$^+$ cells (**D**) in mouse kidneys. Data were presented as mean±SEM and analyzed using one-way ANOVA. N=5:5:5 in each group. *p < 0.05, **p < 0.01.

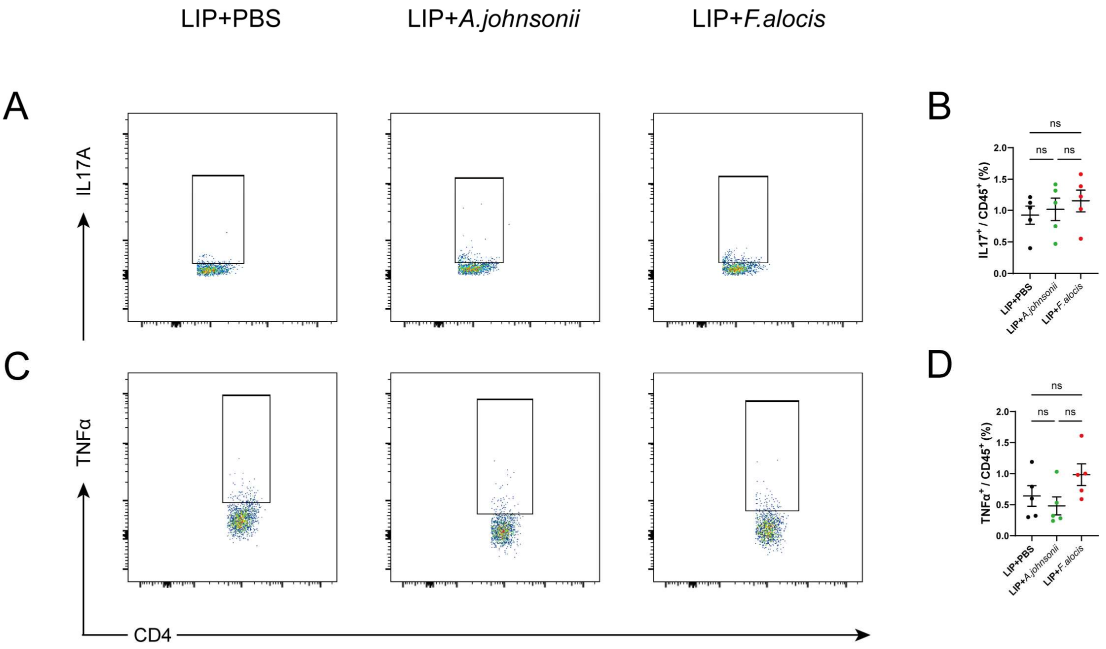

Figure S7. Effect of *Filifactor alocis* on T cell infiltration in aortas of angiotensin ii-infused mice. A, Representative flow cytometry image of CD4$^+$ and CD8$^+$ T cell in the mouse aortas. **B**, Quantification of the percentage of CD4$^+$ and CD8$^+$ T cell in CD45$^+$ cells in the mouse aortas. **C-D**, Representative flow cytometry image of CD4$^+$IFNγ$^+$ T cell (**C**) and quantification of the percentage of CD4$^+$IFNγ$^+$ T cell in CD45$^+$ cells (**D**) in the mouse aortas. **E-F**, Representative flow cytometry image of CD4$^+$IL17A$^+$ T cell (**E**) and quantification of the percentage of CD4$^+$IL17A$^+$ T cell in CD45$^+$ cells (**F**) in the mouse aortas. **G-H**, Representative flow cytometry image of CD4$^+$TNFα$^+$ T cell (**G**) and quantification of the percentage of CD4$^+$TNFα$^+$ T cell in CD45$^+$ cells (**H**) in the mouse aortas. **I-J**, Representative flow cytometry image of CD8$^+$IFNγ$^+$ T cell (**I**) and quantification of the percentage of CD8$^+$IFNγ$^+$ T cell in CD45$^+$ cells (**J**) in the mouse aortas. Data were presented as mean±SEM and analyzed using one-way ANOVA. N=5:5:5 in each group. *p < 0.05, **p < 0.01.

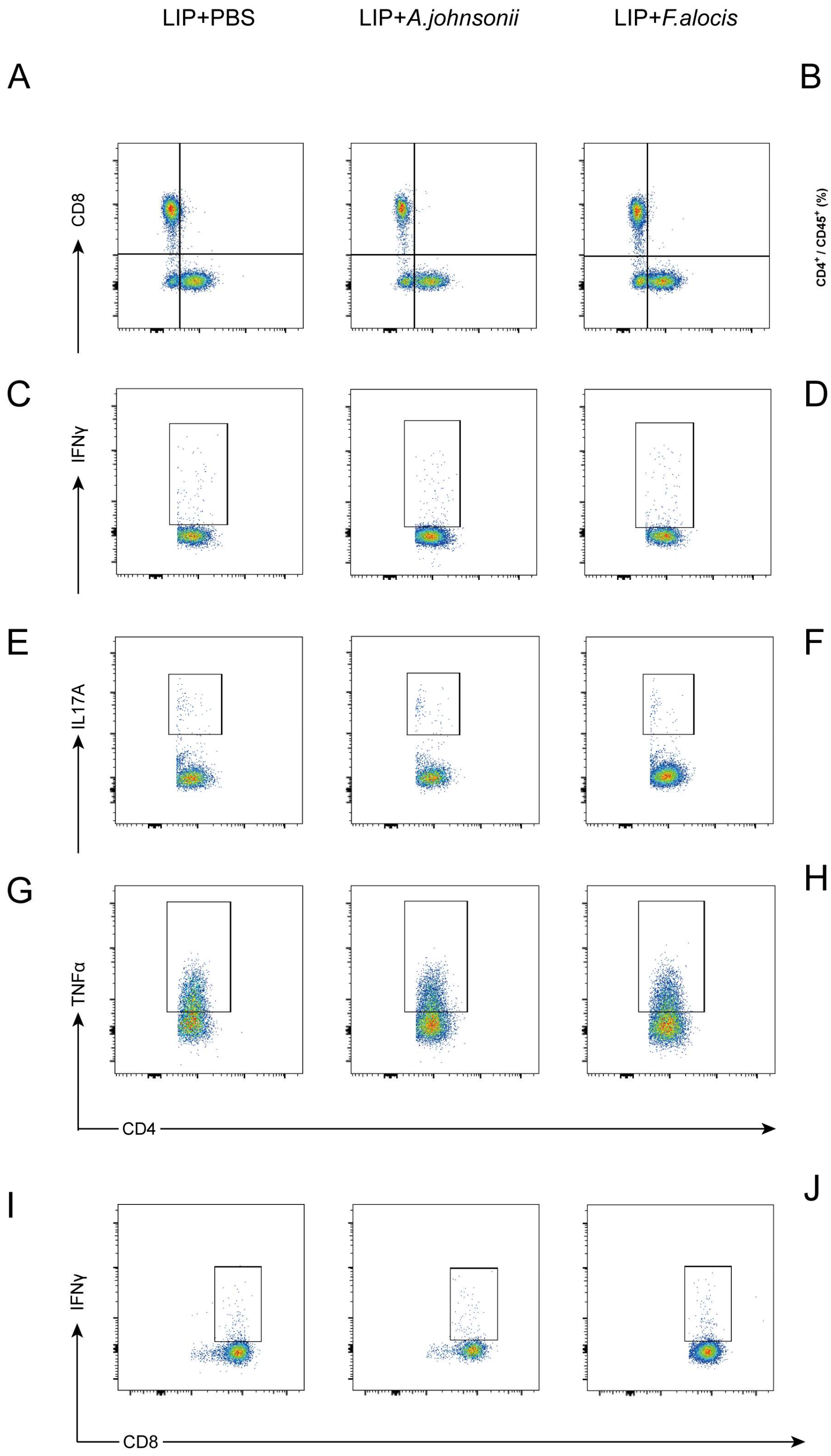

LIP+PBS
LIP+A.johnsonii
LIP+F.alocis
A
B
C
D
E
F
G
H
I
J
CD8
IFNγ
IL17A
TNFα
CD4
IFNγ
CD8
ns
CD4+ / CD45+ (%)
CD8+ / CD45+ (%)
CD4+IFNγ+ / CD45+ (%)
CD4+IL17+ / CD45+ (%)
CD4+TNFα+ / CD45+ (%)
CD8+IFNγ+ / CD45+ (%)
LIP+PBS
LIP+A.johnsonii
LIP+F.alocis
