# Supplementary material for: Linking oral microbiota to periodontitis and hypertension unveils that Filifactor alocis aggravates hypertension via infiltration of interferon-γ+ T cells
Source: mSystems. 2025 May 21;10(6):e00084-25. doi: 10.1128/msystems.00084-25 (PMC12172497; doi:10.1128/msystems.00084-25)
Supplement: Supplemental tables — Tables S1 to S7. [file msystems.00084-25-s0003.docx]

| **Table S1. Demographic of subjects.** | | | | | | |
| --- | --- | --- | --- | --- | --- | --- |
|  |  | **healthy** | **PD** | **HTN** | **PDHTN** | **P value** |
| **Sex** |  |  |  |  |  |  |
|  | **Male** | 4(28.6%) | 3(37.5%) | 8(50%) | 8(42.1%) |  |
|  | **Female** | 10(71.4%) | 5(62.5%) | 8(50%) | 11(57.9%) | 0.6875 |
| **Age** |  | 66.9±9.86 | 68.4±6.95 | 68.5±6.08 | 67.1±5.55 | 0.898 |
| **BMI** |  | 23.4±2.29 | 24.1±2.77 | 23.69±3.20 | 25.17±3.89 | 0.403 |
| **SBP** |  | 120±13.5 | 125±9.75 | 125±16.8 | 134±11 | 0.0414 |
| **DBP** |  | 74.5±6.28 | 77.9±8.32 | 75.1±7.58 | 79.9±8.02 | 0.147 |
| **Probing depth(mm)** |  | ＜4 | ≥4 | ＜4 | ≥4 | NA |
| **Attachment loss(mm)** |  | ＜3 | ≥3 | ＜3 | ≥3 | NA |

HTN, patients with hypertension; PDHTN, patients with hypertension and periodontal disease; SBP, systolic blood pressure; DBP, diastolic blood pressure; periodontitis is defined as the sum of mild, moderate, and severe disease[1].

**Mild periodontitis:** ≥2 interproximal sites with attachment loss (AL) ≥3 mm, and ≥2 interproximal sites with probing depth (PD) ≥4 mm (not on same tooth) or one site with PD ≥5 mm.

**Moderate periodontitis:** ≥2 interproximal sites with AL ≥4 mm (not on same tooth), or ≥2 interproximal sites with PD ≥5 mm (not on same tooth).

**Severe periodontitis:** ≥2 interproximal sites with AL ≥6 mm (not on same tooth) and ≥1 interproximal site with PD ≥5 mm

**Table S2. Primer pairs.**

|  | **5'-3'** |
| --- | --- |
| **Mouse GAPDH forward** | **AGGTCGGTGTGAACGGATTTG** |
| **reverse** | **TGTAGACCATGTAGTTGAGGTCA** |
| **Mouse IL1β forward** | **GCAACTGTTCCTGAACTCAACT** |
| **reverse** | **GCAACTGTTCCTGAACTCAACT** |
| **Mouse TNFα forward** | **CCCTCACACTCAGATCATCTTCT** |
| **reverse** | **GCTACGACGTGGGCTACAG** |
| **Mouse collagen I forward** | **GCTCCTCTTAGGGGCCACT** |
| **reverse** | **ATTGGGGACCCTTAGGCCAT** |
| **Mouse collagen III forward** | **CTGTAACATGGAAACTGGGGAAA** |
| **reverse** | **CCATAGCTGAACTGAAAACCACC** |

| **Table S3. Species-level differential abundance analysis in subgingival samples between HC and PD.** | | | | | | | | | | | | | |
| --- | --- | --- | --- | --- | --- | --- | --- | --- | --- | --- | --- | --- | --- |
| HC VS PD | Prevalence | | LinDA results | | | | |  | MaAsLin2 results | | | | |
|  | N in HC | N in PD | FDR | FC | FC direction | FC Lower | FC Upper | | FDR | FC | FC direction | FC Lower | FC Upper |
| *Pauljensenia hongkongensis* | 14 | 8 | 0.01581 | 4.90237 | - | 0.69389 | 10.4657 |  | 0.01764 | 4.22163 | - | 0.693906 | 9.055791 |
| *Selenomonas sp. oral taxon 478* | 14 | 8 | 0.03859 | 2.87875 | - | 0.76883 | 5.3408 |  | 0.05074 | 2.52369 | - | 0.78848 | 4.6026529 |
| *Selenomonas sp. oral taxon 136* | 14 | 8 | 0.03859 | 2.78966 | - | 0.77283 | 5.12317 |  | 0.05446 | 2.46544 | - | 0.792848 | 4.4922072 |
| *Bergeyella cardium* | 14 | 8 | 0.02269 | 11.9787 | + | 3.30683 | 1.8699 |  | 0.01382 | 16.7886 | + | 4.177924 | 1.9954183 |
| *Treponema putidum* | 14 | 8 | 0.05744 | 5.4667 | + | 1.87307 | 1.59751 |  | 0.04911 | 6.36242 | + | 1.947842 | 1.7112214 |
| *Leptotrichia sp. oral taxon 212* | 14 | 8 | 0.03859 | 3.50232 | + | 1.71695 | 1.36518 |  | 0.02931 | 4.03211 | + | 1.821029 | 1.4415737 |
| *Fusobacterium hwasookii* | 14 | 8 | 0.06093 | 2.76669 | + | 1.39848 | 1.30385 |  | 0.04911 | 3.19785 | + | 1.522016 | 1.3662685 |
| *Aggregatibacter segnis* | 14 | 8 | 0.06093 | 2.73718 | + | 1.37764 | 1.30671 |  | 0.04911 | 3.39034 | + | 1.550502 | 1.3943723 |
| *Leptotrichia buccalis* | 14 | 8 | 0.06093 | 2.72213 | + | 1.35965 | 1.31181 |  | 0.04911 | 3.50892 | + | 1.579953 | 1.4054878 |
| *Campylobacter showae* | 14 | 8 | 0.08035 | 2.01698 | + | 1.18366 | 1.17678 |  | 0.04815 | 2.43151 | + | 1.408488 | 1.2375672 |
|  |  |  |  |  |  |  |  |  |  |  |  |  |  |
| Prevalence refers to the detection frequency of a species among individuals. HC, healthy control; PD, participants with periodontitis; FDR, false discovery rate; FC, fold change; FC direction, "-" means species depleted in PD, "+" means elevated; FC Upper and FC Lower, 95% confidence interval of FC. | | | | | | | | | | | | | |

| **Table S4. Species-level differential abundance analysis in subgingival samples between HC and PDHTN.** | | | | | | | | | | | | | |
| --- | --- | --- | --- | --- | --- | --- | --- | --- | --- | --- | --- | --- | --- |
| HC VS PDHTN |  |  | LinDA results | | | | |  | MaAsLin2 results | | | | |
|  | N in HC | N in PDHTN | FDR | FC | FC direction | FC Lower | FC Upper |  | FDR | FC | FC direction | FC Lower | FC Upper |
| *Pauljensenia hongkongensis* | 14 | 19 | 0.00047 | 4.84676 | - | 2.6527882 | 8.855239 |  | 0.00341 | 4.5335 | - | 2.4718761 | 2.4718761 |
| *Actinomyces sp. oral taxon 171* | 14 | 19 | 0.02521 | 3.33578 | - | 1.5077739 | 7.380044 |  | 0.05857 | 3.14597 | - | 1.4440081 | 1.4440081 |
| *Actinomyces sp. oral taxon 897* | 14 | 19 | 0.0159 | 3.24282 | - | 1.5983835 | 6.579091 |  | 0.04911 | 3.0727 | - | 1.5066553 | 1.5066553 |
| *Actinomyces sp. HMT 175* | 14 | 19 | 0.02956 | 3.19218 | - | 1.442908 | 7.062116 |  | 0.05923 | 3.06235 | - | 1.4146646 | 1.4146646 |
| *Actinomyces oris* | 14 | 19 | 0.02225 | 3.15785 | - | 1.5102667 | 6.602835 |  | 0.05446 | 2.90565 | - | 1.4289673 | 1.4289673 |
| *Actinomyces sp. HMT897* | 14 | 19 | 0.01632 | 3.10704 | - | 1.5605983 | 6.185886 |  | 0.04911 | 2.94446 | - | 1.470302 | 1.470302 |
| *Actinomyces radicidentis* | 14 | 19 | 0.0125 | 3.03651 | - | 1.5984464 | 5.768336 |  | 0.02931 | 2.83391 | - | 1.5675713 | 1.5675713 |
| *Actinomyces viscosus* | 14 | 19 | 0.03833 | 2.95249 | - | 1.3659613 | 6.38174 |  | 0.0728 | 2.75537 | - | 1.3129229 | 1.3129229 |
| *Selenomonas sp. oral taxon 478* | 14 | 19 | 0.0048 | 2.61358 | - | 1.5993051 | 4.271102 |  | 0.01711 | 2.48872 | - | 1.5437486 | 1.5437486 |
| *Streptococcus gordonii* | 14 | 19 | 0.02956 | 2.60804 | - | 1.3577539 | 5.009662 |  | 0.07086 | 2.43476 | - | 1.2757361 | 1.2757361 |
| *Selenomonas sp. oral taxon 136* | 14 | 19 | 0.00929 | 2.41961 | - | 1.4926173 | 3.922301 |  | 0.02931 | 2.3021 | - | 1.4290507 | 1.4290507 |
| *Actinomyces sp. oral taxon 414* | 14 | 19 | 0.01911 | 2.22681 | - | 1.3486455 | 3.676802 |  | 0.04911 | 2.22868 | - | 1.3497953 | 1.3497953 |
| *Bergeyella cardium* | 14 | 19 | 0.00131 | 11.2828 | + | 4.0566616 | 31.38074 |  | 0.00685 | 12.0894 | + | 4.0026876 | 36.513801 |
| *Porphyromonas gingivalis* | 14 | 19 | 0.00209 | 9.20034 | + | 3.4135374 | 24.79722 |  | 0.00685 | 10.0608 | + | 3.5643247 | 28.398254 |
| *Treponema putidum* | 14 | 19 | 0.00282 | 6.04638 | + | 2.5811542 | 14.1637 |  | 0.01382 | 6.73187 | + | 2.6278172 | 17.245496 |
| *Treponema denticola* | 14 | 19 | 0.00282 | 6.03197 | + | 2.5894259 | 14.05125 |  | 0.01382 | 6.66068 | + | 2.5911411 | 17.121662 |
| *Filifactor alocis* | 14 | 19 | 0.00405 | 4.82438 | + | 2.2004024 | 10.57744 |  | 0.01705 | 5.23609 | + | 2.2193889 | 12.35325 |
| *Treponema medium* | 14 | 19 | 0.00302 | 4.33066 | + | 2.1368105 | 8.776916 |  | 0.01705 | 4.78468 | + | 2.1265206 | 10.765535 |
| *Tannerella forsythia* | 14 | 19 | 0.0125 | 3.97812 | + | 1.8023369 | 8.780489 |  | 0.0323 | 4.48351 | + | 1.872471 | 10.735496 |
| *Porphyromonas endodontalis* | 14 | 19 | 0.02242 | 3.5572 | + | 1.566905 | 8.075586 |  | 0.05526 | 3.91719 | + | 1.5708336 | 9.7682859 |
| *Treponema sp. OMZ 838* | 14 | 19 | 0.01049 | 3.29877 | + | 1.6942775 | 6.422736 |  | 0.03948 | 3.55545 | + | 1.6654873 | 7.5901262 |
| *Prevotella intermedia* | 14 | 19 | 0.01445 | 2.71515 | + | 1.5047938 | 4.89903 |  | 0.04911 | 2.93034 | + | 1.4982649 | 5.7312336 |
| *Treponema vincentii* | 14 | 19 | 0.02677 | 2.46868 | + | 1.349957 | 4.514506 |  | 0.06091 | 2.67136 | + | 1.3396311 | 5.3269639 |
| *Campylobacter rectus* | 14 | 19 | 0.01911 | 2.40131 | + | 1.3919253 | 4.142666 |  | 0.02931 | 2.60936 | + | 1.5068173 | 4.5186437 |
|  |  |  |  |  |  |  |  |  |  |  |  |  |  |
| Prevalence refers to the detection frequency of a species among individuals. HC, healthy control; PDHTN, hypertensive participants with periodontitis; FDR, false discovery rate; FC, fold change; FC direction, "-" means species depleted in PDHTN, "+" means elevated; FC Upper and FC Lower, 95% confidence interval of FC. | | | | | | | | | | | | | |

| **Table S5. Species-level differential abundance analysis in salivary samples between HC and PD.** | | | | | | | | | | | | | |
| --- | --- | --- | --- | --- | --- | --- | --- | --- | --- | --- | --- | --- | --- |
| HC VS PD |  |  | LinDA results | | | | |  | MaAsLin2 results | | | | |
|  | N in HC | N in PD | FDR | FC | FC direction | FC Lower | FC Upper |  | FDR | FC | FC direction | FC Lower | FC Upper |
| *Lancefieldella parvula* | 14 | 8 | 0.00339 | 8.6839 | - | 3.4072357 | 22.132368 |  | 0.0026 | 8.69898 | - | 3.432403771 | 22.0464382 |
| *Rothia dentocariosa* | 14 | 8 | 0.02503 | 6.06863 | - | 1.9342673 | 19.039931 |  | 0.02318 | 6.21019 | - | 1.936590721 | 19.9145847 |
| *Prevotella histicola* | 14 | 8 | 0.04655 | 5.85507 | - | 1.5818467 | 21.672052 |  | 0.04582 | 5.86477 | - | 1.60848053 | 21.3838756 |
| *Veillonella atypica* | 14 | 8 | 0.01983 | 5.39889 | - | 2.0132169 | 14.478315 |  | 0.01386 | 5.66452 | - | 2.076953121 | 15.4489591 |
| *Streptococcus sp. LPB0220* | 14 | 8 | 0.02503 | 4.99164 | - | 1.7674108 | 14.097706 |  | 0.02318 | 5.31158 | - | 1.82847512 | 15.4297279 |
| *Streptococcus sp. HSISM1* | 14 | 8 | 0.02503 | 4.67027 | - | 1.7281935 | 12.620945 |  | 0.02375 | 4.93302 | - | 1.77143885 | 13.7372201 |
| *Gemella sanguinis* | 14 | 8 | 0.01983 | 4.5309 | - | 1.8732426 | 10.959082 |  | 0.01706 | 4.5083 | - | 1.831620991 | 11.0966106 |
| *Streptococcus salivarius* | 14 | 8 | 0.0304 | 4.46129 | - | 1.6391098 | 12.142633 |  | 0.02988 | 4.61984 | - | 1.658845731 | 12.8661426 |
| *Schaalia odontolytica* | 14 | 8 | 0.01983 | 4.28436 | - | 1.834453 | 10.006106 |  | 0.01122 | 4.47186 | - | 1.935706069 | 10.3308818 |
| *Actinomyces sp. HMT 175* | 14 | 8 | 0.0255 | 4.26828 | - | 1.6620033 | 10.961578 |  | 0.03289 | 4.25881 | - | 1.58231985 | 11.4625657 |
| *Streptococcus parasanguinis* | 14 | 8 | 0.02503 | 4.1051 | - | 1.6700402 | 10.090692 |  | 0.02241 | 4.298 | - | 1.708030975 | 10.8152479 |
| *Veillonella dispar* | 14 | 8 | 0.03362 | 3.75458 | - | 1.4947428 | 9.4309828 |  | 0.0297 | 3.75217 | - | 1.555976199 | 9.04819286 |
| *Veillonella sp. S12025-13* | 14 | 8 | 0.02503 | 3.72084 | - | 1.5936204 | 8.687554 |  | 0.01822 | 3.86101 | - | 1.700536752 | 8.76629718 |
| *Streptococcus mitis* | 14 | 8 | 0.0334 | 3.60644 | - | 1.490081 | 8.7286551 |  | 0.03432 | 3.60368 | - | 1.491189572 | 8.70884244 |
| *Veillonella nakazawae* | 14 | 8 | 0.02503 | 3.51483 | - | 1.5635446 | 7.9013069 |  | 0.01722 | 3.70212 | - | 1.686829336 | 8.12513269 |
| *Actinomyces oris* | 14 | 8 | 0.04931 | 3.33588 | - | 1.3369643 | 8.3234156 |  | 0.06525 | 3.36537 | - | 1.28406607 | 8.82020129 |
| *Streptococcus pneumoniae* | 14 | 8 | 0.02503 | 2.97068 | - | 1.4912332 | 5.9178672 |  | 0.01969 | 3.00585 | - | 1.520744092 | 5.94125381 |
| *Veillonella parvula* | 14 | 8 | 0.02366 | 2.40891 | - | 1.4196107 | 4.0876404 |  | 0.01684 | 2.43872 | - | 1.437239833 | 4.13802679 |
| *Streptococcus oralis* | 14 | 8 | 0.02503 | 2.18756 | - | 1.3586102 | 3.5222846 |  | 0.01822 | 2.17621 | - | 1.356579919 | 3.49105612 |
| *Fretibacterium fastidiosum* | 14 | 8 | 0.00339 | 13.93 | + | 4.4496152 | 43.609275 |  | 0.00349 | 14.7214 | + | 4.326620485 | 50.0900145 |
| *Bergeyella cardium* | 14 | 8 | 0.02503 | 6.15373 | + | 1.943201 | 19.487645 |  | 0.02988 | 6.19502 | + | 1.822286388 | 21.0605093 |
| *Tannerella forsythia* | 14 | 8 | 0.01454 | 5.50924 | + | 2.306986 | 13.156437 |  | 0.01119 | 5.53864 | + | 2.177646555 | 14.0870149 |
| *Ottowia sp. oral taxon 894* | 14 | 8 | 0.0334 | 4.97874 | + | 1.6365386 | 15.14653 |  | 0.0413 | 5.30081 | + | 1.614711406 | 17.4016257 |
| *Kingella denitrificans* | 14 | 8 | 0.02503 | 4.85402 | + | 1.828293 | 12.887139 |  | 0.02241 | 4.88096 | + | 1.787819445 | 13.3256226 |
| *Porphyromonas endodontalis* | 14 | 8 | 0.01983 | 4.50019 | + | 1.9589064 | 10.338287 |  | 0.0197 | 4.52428 | + | 1.773277206 | 11.5430756 |
| *Treponema denticola* | 14 | 8 | 0.02503 | 4.4376 | + | 1.7556275 | 11.216685 |  | 0.03035 | 4.46131 | + | 1.628553443 | 12.2214702 |
| *Filifactor alocis* | 14 | 8 | 0.03811 | 4.36 | + | 1.5334775 | 12.396407 |  | 0.04993 | 4.38356 | + | 1.451100689 | 13.2420929 |
| *Neisseria macacae* | 14 | 8 | 0.04745 | 3.98747 | + | 1.4206656 | 11.191886 |  | 0.05203 | 4.08864 | + | 1.410299712 | 11.8534979 |
| *Neisseria sicca* | 14 | 8 | 0.03286 | 3.85383 | + | 1.544012 | 9.6191015 |  | 0.03276 | 3.90955 | + | 1.543444527 | 9.90291928 |
| *Treponema medium* | 14 | 8 | 0.02845 | 3.54202 | + | 1.5365203 | 8.1651587 |  | 0.04227 | 3.56083 | + | 1.430596403 | 8.86311407 |
| *Treponema sp. OMZ 838* | 14 | 8 | 0.03301 | 3.2272 | + | 1.4543863 | 7.1609902 |  | 0.04625 | 3.24444 | + | 1.368326305 | 7.69291525 |
| *Neisseria elongata* | 14 | 8 | 0.01983 | 3.05438 | + | 1.6317851 | 5.7171789 |  | 0.01119 | 3.07994 | + | 1.649459049 | 5.75098561 |
| *Prevotella intermedia* | 14 | 8 | 0.02503 | 2.74698 | + | 1.4512107 | 5.1997334 |  | 0.03634 | 2.76135 | + | 1.36350129 | 5.59227076 |
| *Eikenella corrodens* | 14 | 8 | 0.04535 | 2.21585 | + | 1.2355031 | 3.9740792 |  | 0.04735 | 2.22756 | + | 1.232933087 | 4.02458518 |
|  |  |  |  |  |  |  |  |  |  |  |  |  |  |
| Prevalence refers to the detection frequency of a species among individuals. HC, healthy control; PD, participants with periodontitis; FDR, false discovery rate; FC, fold change; FC direction, "-" means species depleted in PD, "+" means elevated; FC Upper and FC Lower, 95% confidence interval of FC. | | | | | | | | | | | | | |

| **Table S6. Species-level differential abundance analysis in salivary samples between HC and HTN.** | | | | | | | | | | | | | |
| --- | --- | --- | --- | --- | --- | --- | --- | --- | --- | --- | --- | --- | --- |
| HC VS HTN |  |  | LinDA results | | | | |  | MaAsLin2 results | | | | |
|  | N in HC | N in HTN | FDR | FC | FC direction | FC Lower | FC Upper |  | FDR | FC | FC direction | FC Lower | FC Upper |
| *Selenomonas sp. oral taxon 478* | 14 | 16 | 0.03745 | 3.54287 | - | 1.82019522 | 6.89592763 |  | 0.01119 | 0.27699 | - | 1.76541834 | 7.3828575 |
| *Leptotrichia sp. oral taxon 221* | 14 | 16 | 0.09288 | 3.36843 | - | 1.59436134 | 7.11654004 |  | 0.02951 | 0.29743 | - | 1.50325988 | 7.51939887 |
| *Selenomonas sp. oral taxon 136* | 14 | 16 | 0.03745 | 3.36093 | - | 1.80030494 | 6.27440341 |  | 0.01119 | 0.29239 | - | 1.7353802 | 6.74047965 |
| *Fretibacterium fastidiosum* | 14 | 16 | 0.03745 | 5.8399 | + | 2.2759046 | 14.9850121 |  | 0.01119 | 6.26586 | + | 2.27960706 | 17.22268 |
| *Porphyromonas gingivalis* | 14 | 16 | 0.03745 | 5.81459 | + | 2.27553577 | 14.8577806 |  | 0.00956 | 6.22277 | + | 2.41198347 | 16.0543622 |
| *Filifactor alocis* | 14 | 16 | 0.05291 | 4.51702 | + | 1.90603717 | 10.7046534 |  | 0.01384 | 4.86629 | + | 1.95318751 | 12.1241634 |
|  |  |  |  |  |  |  |  |  |  |  |  |  |  |
| Prevalence refers to the detection frequency of a species among individuals. HC, healthy control; HTN, hypertensive participants; FDR, false discovery rate; FC, fold change; FC direction, "-" means species depleted in HTN, "+" means elevated; FC Upper and FC Lower, 95% confidence interval of FC. | | | | | | | | | | | | | |

| **Table S7. Species-level differential abundance analysis in salivary samples between HC and PDHTN.** | | | | | | | | | | | | | |
| --- | --- | --- | --- | --- | --- | --- | --- | --- | --- | --- | --- | --- | --- |
| HC VS PDHTN |  |  | LinDA results | | | | |  | MaAsLin2 results | | | | |
|  | N in HC | N in PDHTN | FDR | FC | FC direction | FC Lower | FC Upper |  | FDR | FC | FC direction | FC Lower | FC Upper |
| *Prevotella histicola* | 14 | 19 | 0.00737 | 5.95467 | - | 2.1045782 | 16.8481 |  | 0.01119 | 6.60705 | - | 2.3632304 | 18.471787 |
| *Lancefieldella parvula* | 14 | 19 | 0.00176 | 5.35666 | - | 2.546767 | 11.26675 |  | 0.0026 | 5.94311 | - | 2.8382474 | 12.444486 |
| *Actinomyces sp. oral taxon 169* | 14 | 19 | 0.00531 | 4.87102 | - | 2.0443594 | 11.606 |  | 0.00956 | 5.85118 | - | 2.3488612 | 14.575712 |
| *Streptococcus salivarius* | 14 | 19 | 0.00311 | 4.86911 | - | 2.1971702 | 10.79034 |  | 0.00477 | 5.58528 | - | 2.4747815 | 12.605292 |
| *Prevotella jejuni* | 14 | 19 | 0.00627 | 4.69759 | - | 1.9542225 | 11.29216 |  | 0.0097 | 5.47387 | - | 2.2427262 | 13.360202 |
| *Streptococcus parasanguinis* | 14 | 19 | 0.00311 | 4.13922 | - | 2.0253665 | 8.459286 |  | 0.00449 | 4.80061 | - | 2.3056666 | 9.9953152 |
| *Veillonella atypica* | 14 | 19 | 0.00578 | 4.1033 | - | 1.8735456 | 8.98673 |  | 0.00956 | 4.76893 | - | 2.1484879 | 10.585443 |
| *Actinomyces sp. HMT 175* | 14 | 19 | 0.00454 | 4.09664 | - | 1.9359504 | 8.668842 |  | 0.00956 | 4.52717 | - | 2.0611154 | 9.9437712 |
| *Streptococcus sp. HSISM1* | 14 | 19 | 0.0063 | 4.00886 | - | 1.8192875 | 8.83367 |  | 0.0097 | 4.69039 | - | 2.0783969 | 10.584959 |
| *Streptococcus sp. LPB0220* | 14 | 19 | 0.00946 | 3.89548 | - | 1.706936 | 8.890044 |  | 0.01119 | 4.59126 | - | 1.9672962 | 10.715042 |
| *Actinomyces oris* | 14 | 19 | 0.00531 | 3.76837 | - | 1.8221235 | 7.793445 |  | 0.0105 | 4.21098 | - | 1.9581062 | 9.0558637 |
| *Actinomyces sp. oral taxon 171* | 14 | 19 | 0.00876 | 3.45835 | - | 1.6450432 | 7.270459 |  | 0.0246 | 3.65374 | - | 1.5800849 | 8.4487832 |
| *Schaalia odontolytica* | 14 | 19 | 0.00603 | 3.33744 | - | 1.7008091 | 6.548953 |  | 0.00743 | 3.85868 | - | 1.9835329 | 7.5064955 |
| *Actinomyces viscosus* | 14 | 19 | 0.03317 | 2.91596 | - | 1.2986345 | 6.547514 |  | 0.04656 | 3.21372 | - | 1.3617415 | 7.5843941 |
| *Veillonella dispar* | 14 | 19 | 0.02425 | 2.83056 | - | 1.3614065 | 5.885126 |  | 0.01858 | 3.13337 | - | 1.556708 | 6.3069147 |
| *Veillonella sp. S12025-13* | 14 | 19 | 0.02495 | 2.58466 | - | 1.3174756 | 5.070648 |  | 0.01706 | 2.97086 | - | 1.5483559 | 5.7002504 |
| *Veillonella nakazawae* | 14 | 19 | 0.01896 | 2.58427 | - | 1.3575617 | 4.919434 |  | 0.01208 | 3.01511 | - | 1.6143654 | 5.6312386 |
| *Leptotrichia sp. oral taxon 221* | 14 | 19 | 0.04146 | 2.44686 | - | 1.1911479 | 5.026367 |  | 0.04293 | 2.92909 | - | 1.3498432 | 6.3559643 |
| *Selenomonas sp. oral taxon 478* | 14 | 19 | 0.04219 | 2.21064 | - | 1.1645006 | 4.19657 |  | 0.03892 | 2.65572 | - | 1.3340114 | 5.2869542 |
| *Prevotella scopos* | 14 | 19 | 0.02253 | 2.09436 | - | 1.2524589 | 3.5022 |  | 0.01634 | 2.41222 | - | 1.4351119 | 4.0546141 |
| *Streptococcus oralis* | 14 | 19 | 0.00394 | 2.07034 | - | 1.4178898 | 3.023023 |  | 0.00349 | 2.28141 | - | 1.5670469 | 3.3214171 |
| *Selenomonas sp. oral taxon 136* | 14 | 19 | 0.05127 | 2.03781 | - | 1.1174562 | 3.71619 |  | 0.04582 | 2.44474 | - | 1.2724713 | 4.6969708 |
| *Streptococcus gordonii* | 14 | 19 | 0.03188 | 2.01626 | - | 1.191769 | 3.411153 |  | 0.0413 | 2.24583 | - | 1.2611757 | 3.9992448 |
| *Veillonella parvula* | 14 | 19 | 0.01809 | 1.86915 | - | 1.2278191 | 2.845455 |  | 0.01208 | 2.09604 | - | 1.3769159 | 3.190755 |
| *Streptococcus australis* | 14 | 19 | 0.03322 | 1.75331 | - | 1.1448998 | 2.68504 |  | 0.02392 | 1.93218 | - | 1.2646333 | 2.9521109 |
| *Bergeyella cardium* | 14 | 19 | 0.00053 | 11.0665 | + | 4.4274683 | 27.6606 |  | 0.0026 | 10.8143 | + | 4.0894234 | 28.597719 |
| *Fretibacterium fastidiosum* | 14 | 19 | 0.00053 | 10.9151 | + | 4.4069912 | 27.03416 |  | 0.0026 | 9.93231 | + | 3.7533365 | 26.283484 |
| *Porphyromonas gingivalis* | 14 | 19 | 0.00105 | 8.93049 | + | 3.6202472 | 22.0299 |  | 0.0026 | 8.23518 | + | 3.3076427 | 20.503497 |
| *Neisseria sp. oral taxon 014* | 14 | 19 | 0.00245 | 6.75573 | + | 2.734288 | 16.69168 |  | 0.00956 | 6.19165 | + | 2.4057381 | 15.935454 |
| *Arachnia rubra* | 14 | 19 | 0.01218 | 6.43425 | + | 1.985438 | 20.85164 |  | 0.02988 | 6.2571 | + | 1.8270387 | 21.428841 |
| *Ottowia sp. oral taxon 894* | 14 | 19 | 0.00311 | 5.87095 | + | 2.4249636 | 14.21385 |  | 0.01119 | 5.45638 | + | 2.121451 | 14.033848 |
| *Tannerella forsythia* | 14 | 19 | 0.00105 | 5.46426 | + | 2.7358366 | 10.9137 |  | 0.00349 | 5.10932 | + | 2.4331626 | 10.728888 |
| *Filifactor alocis* | 14 | 19 | 0.00504 | 4.66933 | + | 2.0351814 | 10.71285 |  | 0.01822 | 4.23774 | + | 1.7602156 | 10.202401 |
| *Treponema denticola* | 14 | 19 | 0.00288 | 4.56922 | + | 2.1867311 | 9.547462 |  | 0.01119 | 4.23325 | + | 1.9004486 | 9.4295523 |
| *Morococcus cerebrosus* | 14 | 19 | 0.00364 | 4.38171 | + | 2.0468277 | 9.380046 |  | 0.01119 | 4.27988 | + | 1.9172555 | 9.5539472 |
| *Neisseria macacae* | 14 | 19 | 0.0063 | 4.22124 | + | 1.8588402 | 9.586004 |  | 0.02208 | 3.8313 | + | 1.6442699 | 8.9272789 |
| *Porphyromonas endodontalis* | 14 | 19 | 0.00224 | 4.20625 | + | 2.171842 | 8.146316 |  | 0.01088 | 4.00394 | + | 1.9020315 | 8.4286428 |
| *Kingella denitrificans* | 14 | 19 | 0.00531 | 4.13429 | + | 1.9028115 | 8.982678 |  | 0.01706 | 3.8094 | + | 1.7148013 | 8.4624924 |
| *Neisseria sicca* | 14 | 19 | 0.00828 | 3.40566 | + | 1.6462846 | 7.045254 |  | 0.02671 | 3.09072 | + | 1.4766648 | 6.468995 |
| *Campylobacter rectus* | 14 | 19 | 0.03703 | 3.35891 | + | 1.3003555 | 8.676285 |  | 0.08458 | 3.09675 | + | 1.1789912 | 8.1339696 |
| *Prevotella intermedia* | 14 | 19 | 0.00148 | 3.27754 | + | 1.9738374 | 5.442333 |  | 0.00956 | 3.01361 | + | 1.7200169 | 5.2801004 |
| *Treponema medium* | 14 | 19 | 0.00626 | 3.24931 | + | 1.6731524 | 6.31024 |  | 0.02971 | 2.96392 | + | 1.4359208 | 6.1179126 |
| *Neisseria subflava* | 14 | 19 | 0.02525 | 3.12855 | + | 1.3855905 | 7.064006 |  | 0.0574 | 2.87415 | + | 1.2743505 | 6.4823154 |
| *Treponema sp. OMZ 838* | 14 | 19 | 0.00748 | 2.94763 | + | 1.5645263 | 5.553436 |  | 0.03673 | 2.67626 | + | 1.3475573 | 5.315062 |
| *Aggregatibacter aphrophilus* | 14 | 19 | 0.00311 | 2.9408 | + | 1.7232677 | 5.018537 |  | 0.01208 | 2.77262 | + | 1.5544293 | 4.9454804 |
| *Neisseria cinerea* | 14 | 19 | 0.02495 | 2.68453 | + | 1.3305564 | 5.416317 |  | 0.05908 | 2.44504 | + | 1.2213095 | 4.8949075 |
| *Treponema vincentii* | 14 | 19 | 0.00946 | 2.61452 | + | 1.457108 | 4.691298 |  | 0.04879 | 2.3998 | + | 1.2508836 | 4.6039691 |
| *Parvimonas micra* | 14 | 19 | 0.01304 | 2.59859 | + | 1.4106945 | 4.786764 |  | 0.05203 | 2.38744 | + | 1.2361018 | 4.6111496 |
| *Neisseria meningitidis* | 14 | 19 | 0.03178 | 2.48217 | + | 1.2570839 | 4.901166 |  | 0.0722 | 2.28272 | + | 1.1698103 | 4.4544132 |
| *Capnocytophaga leadbetteri* | 14 | 19 | 0.03061 | 2.45384 | + | 1.2606679 | 4.776288 |  | 0.08975 | 2.25951 | + | 1.1137941 | 4.5837862 |
| *Neisseria elongata* | 14 | 19 | 0.00627 | 2.4147 | + | 1.4672146 | 3.97405 |  | 0.01958 | 2.23409 | + | 1.360104 | 3.6696828 |
| *Capnocytophaga sp. FDAARGO 737* | 14 | 19 | 0.03317 | 2.40796 | + | 1.2385499 | 4.681511 |  | 0.09506 | 2.21716 | + | 1.0954728 | 4.4873828 |
| *Neisseria sp. KEM232* | 14 | 19 | 0.02601 | 2.345 | + | 1.2701858 | 4.329325 |  | 0.07287 | 2.16812 | + | 1.152556 | 4.078534 |
| *Eikenella corrodens* | 14 | 19 | 0.00531 | 2.3269 | + | 1.4627157 | 3.701645 |  | 0.01842 | 2.15966 | + | 1.3496752 | 3.4557434 |
| *Campylobacter showae* | 14 | 19 | 0.03061 | 2.26186 | + | 1.2350402 | 4.14239 |  | 0.09112 | 2.06912 | + | 1.0967724 | 3.9035051 |
| *Neisseria gonorrhoeae* | 14 | 19 | 0.04033 | 2.22123 | + | 1.1743694 | 4.201271 |  | 0.09108 | 2.05933 | + | 1.097241 | 3.8650155 |
| *Fusobacterium nucleatum* | 14 | 19 | 0.01304 | 2.07156 | + | 1.3002014 | 3.30052 |  | 0.07294 | 1.92426 | + | 1.1231243 | 3.2968611 |
|  |  |  |  |  |  |  |  |  |  |  |  |  |  |
| Prevalence refers to the detection frequency of a species among individuals. HC, healthy control; PDHTN, hypertensive participants with periodontitis; FDR, false discovery rate; FC, fold change; FC direction, "-" means species depleted in PDHTN, "+" means elevated; FC Upper and FC Lower, 95% confidence interval of FC. | | | | | | | | | | | | | |

1. Eke PI, Page RC, Wei L, Thornton-Evans G, Genco RJ. Update of the case definitions for population-based surveillance of periodontitis. J Periodontol. 2012;83(12):1449-54; doi: 10.1902/jop.2012.110664.
